# Impact of vaccine herd-protection effects in cost-effectiveness analyses of childhood vaccinations. A quantitative comparative analysis

**DOI:** 10.1371/journal.pone.0172414

**Published:** 2017-03-01

**Authors:** Marisa Holubar, Maria Christina Stavroulakis, Yvonne Maldonado, John P. A. Ioannidis, Despina Contopoulos-Ioannidis

**Affiliations:** 1 Department of Medicine, Division of Infectious Diseases and Geographic Medicine, Stanford University School of Medicine, Stanford, California, United States of America; 2 Department of Pediatrics, Icahn School of Medicine at Mount Sinai/ Elmhurst Hospital Center, New York, New York, United States of America; 3 Department of Pediatrics, Division of Pediatric Infectious Diseases and Department of Health Research and Policy, Senior Associate Dean for Faculty Development and Diversity, Stanford University School of Medicine, Stanford, California, United States of America; 4 Stanford Prevention Research Center, Department of Medicine and Department of Health Research and Policy, Stanford University School of Medicine, Stanford, California, United States of America; 5 Meta-Research Innovation Center at Stanford (METRICS), Stanford University, Stanford, CA, United States of America; University of Louisville School of Medicine, UNITED STATES

## Abstract

**Background:**

Inclusion of vaccine herd-protection effects in cost-effectiveness analyses (CEAs) can impact the CEAs-conclusions. However, empirical epidemiologic data on the size of herd-protection effects from original studies are limited.

**Methods:**

We performed a quantitative comparative analysis of the impact of herd-protection effects in CEAs for four childhood vaccinations (pneumococcal, meningococcal, rotavirus and influenza). We considered CEAs reporting incremental-cost-effectiveness-ratios (ICERs) (per quality-adjusted-life-years [QALY] gained; per life-years [LY] gained or per disability-adjusted-life-years [DALY] avoided), both with and without herd protection, while keeping all other model parameters stable. We calculated the size of the ICER-differences without vs with-herd-protection and estimated how often inclusion of herd-protection led to crossing of the cost-effectiveness threshold (of an assumed societal-willingness-to-pay) of $50,000 for more-developed countries or X3GDP/capita (WHO-threshold) for less-developed countries.

**Results:**

We identified 35 CEA studies (20 pneumococcal, 4 meningococcal, 8 rotavirus and 3 influenza vaccines) with 99 ICER-analyses (55 per-QALY, 27 per-LY and 17 per-DALY). The median ICER-absolute differences per QALY, LY and DALY (without minus with herd-protection) were $15,620 (IQR: $877 to $48,376); $54,871 (IQR: $787 to $115,026) and $49 (IQR: $15 to $1,636) respectively. When the target-vaccination strategy was not cost-saving without herd-protection, inclusion of herd-protection always resulted in more favorable results. In CEAs that had ICERs above the cost-effectiveness threshold without herd-protection, inclusion of herd-protection led to crossing of that threshold in 45% of the cases. This impacted only CEAs for more developed countries, as all but one CEAs for less developed countries had ICERs below the WHO-cost-effectiveness threshold even without herd-protection. In several analyses, recommendation for the adoption of the target vaccination strategy depended on the inclusion of the herd protection effect.

**Conclusions:**

Inclusion of herd-protection effects in CEAs had a substantial impact in the estimated ICERs and made target-vaccination strategies more attractive options in almost half of the cases where ICERs were above the societal-willingness to pay threshold without herd-protection. More empirical epidemiologic data are needed to determine the size of herd-protection effects across diverse settings and also the size of negative vaccine effects, e.g. from serotype substitution.

## Introduction

Cost effectiveness analysis (CEA) studies [1] have been increasingly used worldwide and in the US in particular [2,3] for the development of national immunization strategies. CEA conclusions can be affected by different methodological choices, modeling choices and populations targeted. Baseline vaccine efficacy assumptions can be influential [4–12]. Of particular interest is the potential impact of indirect vaccine effect assumptions, and specifically vaccine herd-protection (positive effect) and serotype substitution (negative effects) in the community. Herd-protection is the reduction of the disease in non-vaccinated susceptible individuals from widespread humoral immunity and/or decreased carriage (e.g. nasopharyngeal carriage) in vaccinated individuals in the community, that lead to decreased likelihood of non-vaccinated individuals having contact with infected/infectious individuals [13]. Although this phenomenon is widely described, empirical epidemiologic data on the size of indirect vaccine effects are limited. Vaccine CEAs that include indirect vaccine effects in their analyses either use modeling or extrapolate data from studies conducted in other countries, which may have different disease epidemiology.

Vaccine herd-protection has been reported for several childhood vaccinations including pneumococcal (e.g. PCV7, PCV10 and PCV13) [13–18] meningococcal, [11,19–21] rotavirus [22,23] and influenza vaccines. [24,25] We evaluated the overall impact of including herd-protection assumptions in CEAs for these four childhood vaccinations. We addressed the following questions: How often does the inclusion of herd-protection change the conclusions of CEAs and give favorable results for the target vaccination strategy? How large is the impact of including herd-protection in CEAs? How often does the inclusion of herd-protection drive estimates below the willingness-to pay cost-effectiveness-thresholds? Is there a consistent pattern of herd-protection impact across these four vaccines? And finally, is the impact of herd-protection larger in CEAs for more-developed countries and when industry is involved?

## Methods

### Inclusion and exclusion criteria

In this evaluation we considered economic analyses characterized by their authors as CEAs, cost-utility analyses or cost-benefit analyses. We will use the term CEA for consistency unless stated otherwise. We included analyses published in English that targeted childhood vaccination strategies for pneumococcal, meningococcal, rotavirus or influenza disease in infants, children or adolescents. Analyses of strategies vaccinating only adults were excluded. We further considered those studies that reported incremental cost effectiveness ratios (ICER) between the target vaccination strategy and the comparator vaccination strategy (or no vaccination) both with and without herd-protection. CEAs that included herd-protection in their base-case scenario (or primary model) were considered eligible if they also performed separate sensitivity/subgroup/sub-model/scenario analyses without herd-protection. Analyses that reported only cost-effectiveness ratios for single vaccination strategies, and not incremental cost-effectiveness ratios between compared vaccination strategies, were excluded. Data for composite indirect vaccine effects including both herd-protection (positive effect) and serotype substitution (negative effect) or only serotype substitution were not included in our primary analyses (data were very limited to allow for meaningful separate analyses).

Our primary ICER metric was the ICER per quality adjusted life-years gained (QALYs), which was the ratio of the incremental cost divided by the QALYs gained (ICER per-QALYs) by the target vaccination strategy versus the comparator vaccination strategy (or no vaccination). We also considered as secondary metrics the ICER per life-years gained (ICER per-LYs) and ICER per disability adjusted life-years avoided (ICER per-DALYs).

### Search strategy

We searched PubMed and the Tufts CEA registry [26] (last search was January 1, 2014; search-strategy in Text A in S1 File). For the Tufts CEA Registry searches we entered the type of vaccine (i.e. influenza vaccine) into the basic search function. We also screened the reference list of prior systematic reviews of CEA for the four targeted childhood vaccinations. Reviews, commentaries, editorials, letters, abstracts from meetings and articles published in non-English language were excluded. Articles were screened at Title/Abstract level and potentially eligible articles were reviewed in full text.

### Data extraction

From each eligible study we extracted the following information: first author; journal; year; compared interventions (target vaccination strategy versus comparator vaccination strategy or no vaccination [for the characterization of vaccination strategies as target or comparator we used the authors’ definitions and if this was unclear, we considered as target the most recently approved vaccine]); perspective for the cost-analysis (societal or health care system); model (static vs dynamic model [dynamic models are able to produce empiric results influenced by herd-protection indirect-vaccine-effects while static models rely on assumptions for herd-protection]); target population (cohort model vs population model); vaccination coverage rates; monetary unit used (currency and year); industry involvement; size of assumed/modeled herd-protection effect (and reference(s) cited thereof); quantitative ICERs with and without herd-protection per-QALYs gained, per-LYs gained, and per-DALYs avoided (between the compared vaccination strategies; [negative ICERs indicated cost-saving with the target vaccination strategy vs. the comparator]) and the authors’ interpretation of the CEAs findings (the target vaccination strategy was recommended, not recommended, or statement was unclear). Pertinent quantitative data presented only in figures were also extracted using the WebPlot Digitizer software [27]. Estimates in foreign currencies were converted to US dollars for the same year using the OANDA’s currency calculator tool [28]. All values were subsequently inflated to 2016 US dollars to allow comparability of results [29].

When CEAs studies reported analyses for different pairs of compared vaccination strategies, perspectives (e.g. societal or healthcare) and/or for different countries, we considered these as separate analyses.

### Scenarios considered

For our “without herd-protection” analyses we used the base-case scenario if it was clearly defined as without herd-protection. If the base-case scenario was not clearly defined, we selected the scenario that was without herd-protection and had the least number of additional assumptions for other parameters (e.g. for discount rates, vaccination coverage rates, waning vaccination immunity, etc.). We considered studies using either static models (provided that sensitivity analyses with herd-protection were also considered in addition to analyses without herd-protection) or dynamic models. If the study used a dynamic model and the base-case scenario already included herd-protection, we selected sensitivity/subgroup/sub-model/or scenario analyses that were “without herd-protection” and had the same assumptions for all other parameters as the base-case scenario.

For the analyses “with herd-protection” (if multiple scenarios were reported), we always selected the scenario closest, in terms of additional parameter assumptions, to the base-case scenario without herd-protection. In 3 pneumococcal-conjugate vaccine [30–32] and 1 rotavirus vaccine study [33] where different herd-protection assumptions were considered, we decided a priori to keep the analyses for a herd-protection rate closest to 15%. Moreover, we considered only analyses that included herd-protection for both of the compared vaccination strategies.

Two reviewers (MCS, MH) independently extracted data and discrepancies were further evaluated by a third reviewer (DCI) and solved by consensus.

### Statistical analysis

For each compared vaccination strategies we calculated the absolute differences of ICERs per-QALYs gained, of ICERs per-LY gained and of ICERs per-DALYs avoided, “without herd-protection” minus “with herd-protection.” A difference with a positive value indicates that the ICER with herd-protection was more favorable than the ICER without herd-protection. We evaluated the pattern of impact of herd-protection in CEAs across different ICER-metrics and across difference vaccines (median and inter-quartile ranges of ICERs’ differences) and compared ICER-differences across metrics and across vaccines by the non-parametric Kruskal-Wallis test.

In cases where the comparator-strategy was no vaccination we also calculated how often the inclusion of herd-protection led to crossing the cost-effectiveness threshold, from an ICER above that threshold without herd-protection to an ICER below that threshold with herd-protection [34]. For more-developed countries a threshold of $50,000 (or ~£30,000 respectively), representing the assumed societal willingness-to-pay for a QALY (or LY) gained, is often used [35–38]; while for less-developed countries the WHO-cost-effectiveness-threshold of X3GDP/capita (gross domestic product per capita) is often used (Text B in S1 File) [39]. In exploratory analyses, we also evaluated whether the number of ICER-analyses crossing the cost-effectiveness threshold (without vs with herd-protection) differed according to country setting (more-developed versus less-developed countries, as defined in Figure A in S1 File); industry involvement; perspective (societal versus health care) and model (dynamic vs static).

We also captured how often the authors concluded that the target vaccination strategy would be considered cost-effective only if herd-protection was also taken into account.

## Results

### Characteristics of included CEAs

**Fig 1:**
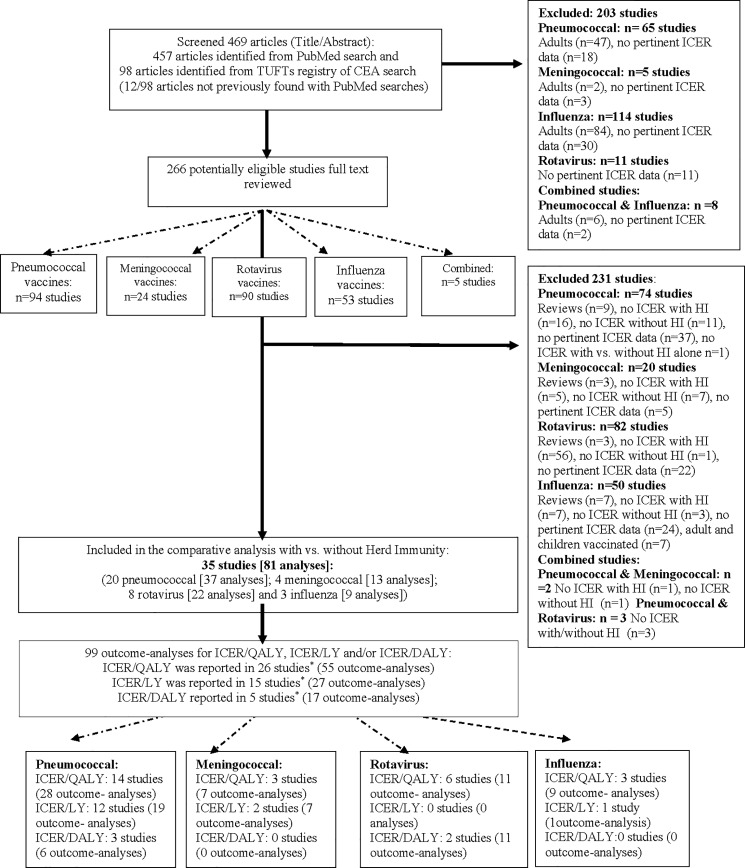
Flow chart. Additional information in Text D in S1 File.

We screened 469 articles and identified 35 eligible studies [30–33; 40–70] (20 for pneumococcal conjugate vaccines; 4 for meningococcal vaccines (3 conjugate vaccines and 1 polysaccharide vaccines); 8 for rotavirus and 3 for influenza-virus vaccines) with a total of 81 separate analyses for different compared vaccination strategies, countries, and/or perspectives (37 pneumococcal, 13 meningococcal, 22 rotavirus and 9 influenza vaccination strategies analyses); and a total of 99 ICER-analyses (n = 55 per-QALYs [26 studies]; n = 27 per-LYs [15 studies] and 17 per-DALYs [5 studies] (Fig 1, Table 1 and Table A in S1 File). Each study could have reported more than one of these ICERs.

**Table 1:** Characteristics of CEA studies (n = 35).

Vaccine	Author	Year	Compared vaccination strategies	Country (ies)	Perspect-ive	Industry involve-ment	ICER/ QALY gained analyses	ICER/LY gained analyses	ICER/ DALY averted analyses
P	Bergman A., et al	2008	1 (PCV 7, 2+1 doses VS no vaccination)	Sweden	S	Yes	1	1	0
P	Blank, P. R. & Szucs, T. D.	2012	1 (PCV 13, 2+1 doses VS PCV7, 2+1 doses)	Switzerland	H	Yes	1	0	0
P	Chuck Anderson W, et al.	2010	2 (Phid-10 VS PCV7 3+1 doses; Phid-10-N vs PCV13)	Canada	H	Yes	2	0	0
P	Díez-Domingo J, et al.	2011	1 (PCV 13, 2+1 doses VS no vaccination)	Spain	H	Yes	1	0	0
P	Earnshaw, S. R., et al.	2012	1 (PCV 13, 2+1 doses VS PCV 10, 2+1 doses)	Canada	H	Yes	1	1	0
P	Giglio N.D. et al	2010	1 (PCV 7, 3+1 doses VS no vaccination)	Argentina	S	Yes	0	1	0
P	Gomez J.A. et al	2013	1 (PCV10 2+1 VS no vaccination)	Peru	H	Yes	1	0	0
P	Hoshi, S. L., et al.	2012	2 (PCV-7, 3+1 doses (Vaccine alone [1000 Y co-pay] VS no vaccination; PCV-7, 3+1 doses (Co-vaccine [1000 Y co-pay] VS no vaccination)	Japan	S	No	2	2	0
P	Hubben, G.A.A., et al	2007	1 (PCV 7, 4 doses VS no vaccination)	Netherlands	S	No	1	1	0
P	Kim SY et al	2010	3 (PCV 7, 3 doses; PCV 9/PCV 10, 3 doses; PCV 13, 3 doses VS no vaccination)	Gambia	S	No	0	0	3
P	Marti SG. et al.	2013	1 (PCV10 3+1 VS no vaccination)	Argentina, Peru, Chile, Colombia, Brazil, Mexico	H	Yes	6	6	0
P	McIntosh E.D.G., et al.	2005	1 (PCV 7, 4 doses VS No vaccination)	UK	S	Yes	0	1	0
P	Melegaro A et al.	2004	1 (PCV 7, 3+1 doses VS no vaccination)	England & Wales	H	No	1	1	0
P	Newall AT, et al.	2011	4 (PCV10 (3+1) VS PCV 7 (3+0); PCV 13 (3+0) VS PCV 7 (3+0); PCV 10 (3+1) VS no vaccination; PCV 13 (3+0) VS no vaccination))	Australia	H	Yes	4	0	0
P	Ray G.T., et al.	2006	1 (PCV7 VS no vaccination)	USA	NR	Yes	0	1	0
P	Rubin J.L, et al	2011	1 (PCV 13, 4 doses VS PCV 7, 4 doses)	USA	S	Yes	1	1	0
P	Tyo K.R., et al	2011	3 (PCV 13, 3 doses VS no vaccination, PCV 7, 3 doses VS no vaccination, Phid10, 3 doses VS no vaccination)	Singapore	H	Yes	3	0	0
P	Uruena A., et al.	2011	2 (PCV 10, 3+1 VS no vaccination; PCV 13, 3+1 VS no vaccination)	Argentina	H	Yes	0	0	2
P	Vespa G., et al.	2009	1 (PCV7, 2+1 VS no vaccination)	Brazil	S	Yes	0	1	1
P	Wisløff T, et al.	2006	2 (PCV 7,3+1 doses VS no vaccination, PCV 7, 2+1 doses VS no vaccination)	Norway	S	Yes	2	2	0
M	Christensen H. et al.	2013	4 (MenB (3+1 @2,3,4 + 12 m; 3+1 @ 2,4,6 + 12 m; 4+1 @2,3,4 + 12 m + catch up 1-4y; 4+1 @2,3,4 + 12 mo + catch up 1-17y VS no vaccination)	UK	H	No	4	0	0
M	De Wals, P. & Erickson, L.	2002	1 (Men C mass immunization, 1 dose VS no vaccination)	Canada	S+H	No	2	2	0
M	Hepkema H. et al.	2013	2 (MenACWY @14m +MenACWY @ 12y VS MCV @ 14m; MenACWY @14m +MenACWY @ 12y VS MenACWY @ 14m)	Netherlands	S	Yes	2	0	0
M	Trotter, C. L., & Edmunds, W. J.	2006	6 (MCV-C, 3 doses; MCV-C, 1 dose; MCV-C, 3+1 (booster <18y); MCV-C, 3+1 (booster <25y); MCV-C, 1+1; MCV-C 2 doses VS no vaccination)	England & Wales	H	No	0	6	0
R	Atherly, D. E., et al.	2012	1 (Rotavirus vaccine, 2 doses VS no vaccination)	Central/South America, Europe, Africa, Eastern Mediterranean, SE Asia, W Pacific, All GAVI	H	No	0	0	7
R	Atkins, K. E., et al.	2012	2 (Rotateq, 3 doses (concomitantly–alligned with UK vaccination schedule; Rotateq, 3 doses separately not–alligned with UK vaccination schedule VS no vaccination)	England & Wales	H	Yes	2	0	0
R	Bakir, M., et al.	2013	1 (Rotarix, 2 doses VS no vaccination)	Turkey	H	Yes	1	0	0
R	Bruijning-Verhagen P. et al.	2013	1 (universal RV VS no vaccination)	Netherlands	H	No	1	0	0
R	Jit, M., et al.	2009	1 (Rotarix, 2 doses VS RotaTeq,3 doses)	Belgium, England &Wales, Finland, France, Netherlands	H	Yes	5	0	0
R	Mangen, M. J., et al.	2010	2 (Rotarix, 2 doses; Rotateq, 3 doses VS no vaccination)	Netherlands	S+H	No	0	0	4
R	Rozenbaum, M. H., et al.	2011	1(RV in national immunization program, 3 doses (€75 cost) VS no vaccination)	Netherlands	S	Yes	1	0	0
R	Tu, H. A., et al.	2013	1 (RotaTeq, 3 doses VS no vaccination)	Netherlands	S	Yes	1	0	0
F	Clements, K. M., et al.	2011	1 (Universal seasonal flu mass vaccination VS targeted seasonal flu vaccination)	USA	S	Yes	1	1	0
F	Newall AT. et al.	2013	1 (TAIV @ 5-17y VS current vaccination practice)	Australia	S+H	No	2	0	0
F	Pitman, R. J., et al.	2013	7 (Current Practice of vaccinating those at increased risk of influenza associated morbidity with TIV; Current Practice + TIV in 2–4 yrs; Current Practice + LAIV in 2–4 yrs; Current Practice + TIV in 2–10 yrs; Current Practice + LAIV in 2–10 yrs; Current Practice + TIV in 2–18 yrs; Current Practice + LAIV in 2–18 yrs; VS no vaccination)	England & Wales	H	Yes	7	0	0

Citations for included studies = refs [35–69]. **Abbreviations:** H: healthcare perspective; ICER/QALY: number of ICER/QALY analyses per study; ICER/LY: number of ICER/LY analyses per study; ICER/DALY: number of ICER/DALY analyses per study; LAIV: live attenuated influenza vaccine; MCV: meningococcal C conjugate vaccine; Men B: meningococcal B conjugate vaccine; NR: not reported; PCV: pneumococcal conjugate vaccine; PHid10:10-valent pneumococcal conjugate vaccine, conjugated to *Hemophilus influenzae* protein-D; S: societal perspective; TIV: trivalent inactivated influenza vaccine.

Industry was involved in 69% (24/35) of the CEA studies (and in 64% [63/99] of ICER-outcome-analyses respectively) (Table 1 and Table A in S1 File); the healthcare perspective was analyzed in 70% (69/99) of ICER-outcome-analyses and static models were used in 83% (29/35) of the CEA studies (and 73% [72/99] of ICER-outcome analyses respectively). Additional characteristics of the included studies and ICER-analyses, including the models, assumed vaccination coverage rates and herd-protection assumptions, are described in detail in Table 1 and Tables A- C in S1 File.

In 16% (16/99) of ICER-analyses the target vaccination strategy was already cost saving even without herd-protection (Table 2). In 35% (6/17) of ICERs per-DALYs analyses the estimates without herd-protection were <$150.

**Table 2:** Differences in ICER/QALY, ICER/LY and ICER/DALY with vs. without Herd protection*.

Vaccine-Country	Author	Comparator	Difference ICER/QALY (Inflated to USD 2016)	ICER/ QALY Without HP (Inflated to USD 2016)	ICER/ QALY With HP (Inflated to USD 2016)	Difference ICER/LY (Inflated to USD 2016)	ICER/LY Without HP (Inflated to USD 2016)	ICER/LY With HP (Inflated to USD 2016)	Difference ICER/ DALY (without vs with HP) (Inflated to USD 2016)	ICER/ DALY Without HI (Inflated to USD 2016)	ICER/ DALY With HI (Inflated to USD 2016)
PCV7-Sweden	Bergman A et al.	No vaccine	36,816.9	45,355.71	8,538.82	66,205	75,891	9687			
PCV7,PCV13-Switzerland	Blank PR et al.	PCV7 (2+1)	28,108.	22,314	5,794						
PCV10, -Canada	Chuck AW et al.	PCV13	15,620	-20,301	-35,920						
PCV10-Canada	Chuck AW et al.	PCV7 (3+1)	-3,001	-36,175	-33,174						
PCV13-Spain	Díez-Domingo J et al.	No vaccine	39,955	44,105	4,150						
PCV13-Canada	Earnshaw SR et al.	PCV 10 (2+1)	- 12,455	-23,650	-11,196	-12,709	-22,456	-9,736			
PCV7-Argentina	Giglio ND et al.	No vaccine				3,177	6,403	3,226			
PCV10-Peru	Gomez JA et al.	No vaccine	1,062	4,974	3,911						
PCV7-Japan	Hoshi SL et al.	No vaccine	53,353	98,829	45,477	129,405	239,950	110,545			
PCV7 co-vaccinate-Japan	Hoshi SL et al.	No vaccine	53,353	98,829	45,477	129,405	239,950	110,545			
P-Netherlands	Hubben GAA et al.	No vaccine	44,648	66,504	21,856	67,284	91,637	24,354			
PCV7-Gambia	Kim SY et al.	No vaccine							49	813	765
PCV9/10-Gambia	Kim SY et al.	No vaccine							73	595	523
PCV13-Gambia	Kim SY et al.	No vaccine							49	498	449
PCV10-Argentina	Martí SG et al.	No vaccine	59	3,700	3,642	771	15,625	14,854			
PCV10-Brazil	Martí SG et al.	No vaccine	877	7,807	6,930	1,064	8,874	7,810			
PCV10-Chile	Martí SG et al.	No vaccine	6	-253	259	-317	-4,184	-3,864			
PCV10-Colombia	Martí SG et al.	No vaccine	149	4,428	4,280	449	9,180	8,731			
PCV10-Mexico	Martí SG et al.	No vaccine	496	5,059	4,564	787	6,359	5,571			
PCV10-Peru	Martí SG et al.	No vaccine	71	3,288	3,217	237	7,075	6,837			
P-UK	McIntosh EDG et al.	No vaccine				58,093	66,728	8,637			
P-UK	Melegaro A et al.	No vaccine	108,811	118,741	9,930	213,801	224,293	10,493			
P-Australia	Newall AT et al.	PCV 7 (3+0)	3,546	21,143	17,597						
P-Australia	Newall AT et al.	No Vaccine	11,896	48,379	36,483						
P-Australia	Newall AT et al.	No Vaccine	3,511	43,898	40,387						
P-Australia	Newall AT et al.	PCV 7 (3+0)	17,316	29,465	12,150						
P-Australia	Newall AT et al.	No Vaccine	16,629	74,380	57,751						
P-USA	Ray GT et al.	No vaccine				131,170	140,585	9,414			
P-USA	Rubin JL et al.	PCV 7 (4 doses)	12,942	7600	20,542	2998	-21,496	-18,499			
P-Singapore	Tyo KR et al.	No vaccine	205,185	252,242	47,057						
P-Singapore	Tyo KR et al.	No vaccine	212,876	261,918	49,041						
P-Singapore	Tyo KR et al.	No vaccine	181,475	222,408	40,934						
PCV-10-Argentina	Uruena A et al.	No vaccine							1,636	9,917	8,281
PCV-13-Argentina	Uruena A et al.	No vaccine							1,432	12,100	10,668
P-Brazil	Vespa G et al.	No vaccine				54,871	81,897	27,027	1,603	2,392	789
P-Norway	Wisløff T et al.	No vaccine	68,689	218,556	149,867	246,657	485,508	238,851			
P-Norway	Wisløff T et al.	No vaccine	40,589	129,573	88,984	146,745	287,246	140,501			
M-UK	Christensen H et al.	No vaccine	136,472	332,601	196,129						
M-UK	Christensen H et al.	No vaccine	147,709	335,257	187,547						
M-UK	Christensen H et al.	No vaccine	287,858	487,256	199,397						
M-UK	Christensen H et al.	No vaccine	422,085	592,471	170,386						
M-Canada	De Wals P et al.	no vaccine	48,376	110,753	62,377	59,832	133,667	73,834			
M-Netherlands	Hepkema H et al.	MCC (@ 14m)	261,156	540,176	279,019						
M-Netherlands	Hepkema H et al.	MenACWY(@14m)	359,334	751,509	392,175						
M-UK	Trotter CL et al.	No vaccine				27,792	34,703	6,911			
M-UK	Trotter CL et al.	No vaccine				75,029	93,225	18,196			
M-UK	Trotter CL et al.	No vaccine				75,432	94,131	18,699			
M-UK	Trotter CL et al.	No vaccine				23,604	32,836	9,232			
M-UK	Trotter CL et al.	No vaccine				44,502	67,232	22,731			
M-UK	Trotter CL et al.	No vaccine				115,026	194,728	79,702			
R-All GAVI	Atherly DE et al.	No vaccine							10	46	36
R-SEAR	Atherly DE et al.	No vaccine							15	65	50
R-EUR	Atherly DE et al.	No vaccine							41	126	85
R-WPR	Atherly DE et al.	No vaccine							45	251	207
R-AMR	Atherly DE et al.	No vaccine							14	68	54
R-AFR	Atherly DE et al.	No vaccine							9	41	33
R-EMR	Atherly DE et al.	No vaccine							5	33	27
R-UK	Atkins KE et al.	No vaccine	12,809	92,178	79,368						
R-UK	Atkins KE et al.	No vaccine	12,810	58,574	45,764						
R-Turkey	Bakir M et al.	No vaccine	-27	-12592	-12562						
R-Netherlands	Bruijning-Verhagen	No vaccine	18,643	88,372	69,728						
R-France	Jit M. et al.	RotaTeq	-17,079	-41,473	-24,394						
R-Finland	Jit M. et al.	RotaTeq	-12,198	-29,273	-17,075						
R-Netherlands	Jit M. et al.	RotaTeq	- 17,078	- 26,835	- 9,757						
R-UK	Jit M. et al.	RotaTeq	-26,835	-80,504	-53,670						
R-Belgium	Jit M. et al.	RotaTeq	- 12,199	-21,955	- 9,756						
R-Netherlands	Mangen MJ et al.	No vaccine							3,373	85,626	82,253
R-Netherlands	Mangen MJ et al.	No vaccine							13,581	72,361	58,780
R-Netherlands	Mangen MJ et al.	No vaccine							6,739.	78,268	71,528
R-Netherlands	Mangen MJ et al.	No vaccine							3,432	79,745	76,313
R-Netherlands	Rozenbaum MH et al.	No vaccine	26,471	67,452	40,980						
R-Netherlands	Tu HA et al.	No vaccine	17,904	22,524	4,620						
F-USA	Clements KM et al.	targeted vaccination	37,227	-101,059	- 138,26	32,121	-79,907	- 112,029			
F-Australia	Newall AT et al.	Current practice	47,159	50,735	3,576						
F-UK	Newall AT et al.	Current practice	47,139	36,682	-10,457						
F-UK	Pitman RJ et al.	No vaccine	4,747	3,894	-852						
F-UK	Pitman RJ et al.	No vaccine	4,151	3,283	-868						
F-UK	Pitman RJ et al.	No vaccine	8,020	7,351	-668						
F-UK	Pitman RJ et al.	No vaccine	7,405	6,717	-688						
F-UK	Pitman RJ et al.	No vaccine	10,012	9,463	-548						
F-UK	Pitman RJ et al.	No vaccine	9,667	9,091	-576						

**Abbreviations:** MCC: meningococcal C conjugate vaccine; MenACWY: meningococcal ACWY conjugate vaccine; PCV7: 7-valent pneumococcal conjugate vaccine; PCV10: 10-valent pneumococcal conjugate vaccine

* Values are in US dollars (All values were inflated to reflect values in 2016 US dollars [29])

† In all cases where the difference without vs with HP was negative (8 ICER/QALY [37, 39, 63] and 3 ICER/LY [39, 45, 50] outcome-analyses); the experimental strategy was already cost-saving without inclusion of indirect effects and with inclusion of indirect effects it was still cost saving (based on absolute cost), although the ratio ICER/QALY with vs without herd immunity was not incrementally more favorable.

Furthermore, among the analyses that were already cost saving without herd immunity (12 ICER/QALY and 4 ICER/LY outcome-analyses); inclusion of herd immunity gave additionally more favorable results in 4 ICER/QALY outcome-analyses [37, 45, 50, 68] and 1 ICER/LY [68] outcome-analyses.

### Incremental effects with herd-protection (n = 99 ICER-analyses)

The median absolute ICER-differences without vs with herd-protection were $15,620 (IQR: $877 to $48,376; range $-35,835 to $422,085) for ICERs per-QALYs; $54,871 (IQR: $787 to $115,026; range $-12,719 to $246,657) for ICERs per-LYs; and $49 (IQR: $15 to $1,636; range $5 to $13,581) respectively for ICERs per-DALYs (Table 2, Table E in S1 File and Figure B-2 in S1 File).

**Fig 2:**
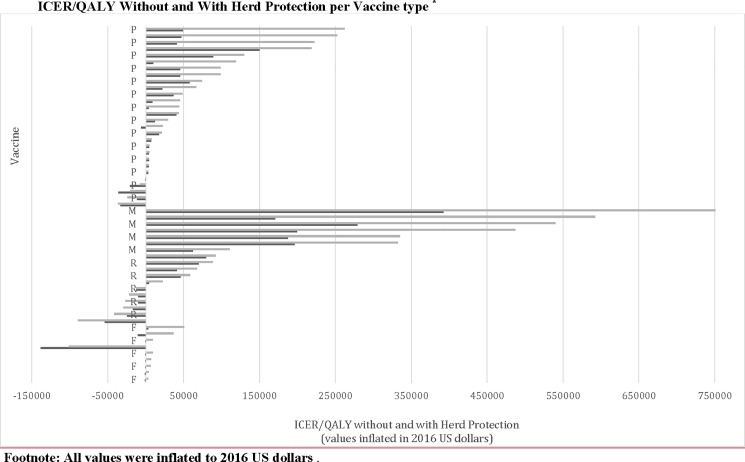
Barplot of ICER per-QALYs without vs. with herd-protection across all four childhood vaccines. X-axis: ICERs per-QALY with herd protection (values inflated to 2016 US dollars, [29]); Grey bars: ICERs per-QALY without Herd Protection; Black bars: ICERs per-QALY with herd-protection. **Abbreviations**: P = pneumococcal vaccines, M = meningococcal vaccines, R = rotavirus vaccines, F = influenza vaccines.

The bar-plot of the differences across all 4 vaccines of ICERs per-QALYs, with vs without herd-protection, are shown in Fig 2 and Figure C in S1 File; and of the differences of ICERs per-LYs and ICERs per-DALYs in Figure D in S1 File.

Across all vaccines, inclusion of herd-protection gave more favorable results in 89% (88/99) of ICER-analyses (85% of ICER per-QALYs; 89% of ICER per-LYs and in 100% of ICER per-DALYs analyses) (Table D in S1 File). In the remaining 11/99 ICER-analyses the target-vaccination strategies were already cost saving without herd-protection.

When the target vaccination strategies were not already cost saving without herd-protection (n = 83/99), ICERs were always more favorable with inclusion of herd-protection (Table 2 and Table D in S1 File). Among the 16 ICER-analyses that were already cost saving without herd-protection (12 ICER per-QALYs and 4 ICER per-LYs analyses), inclusion of herd-protection gave additionally more favorable cost-saving results in 31% (5/16) of those.

### Crossing of cost-effectiveness thresholds

Overall, among 79 ICER-analyses that compared target vaccine strategies vs no vaccination, 48% (38/79) had ICERs that without herd-protection were above the cost-effectiveness threshold of $50,000 for more developed countries and X3GDP/capita for less developed countries (37 ICER-analyses for more-developed countries and 1 ICER-analysis for a less-developed country) (Table D in S1 File). (The WHO-thresholds of X3GDP/capita reported in individual studies for less developed countries are shown in Text B in S1 File).

In 45% (17/38) of those ICERs that were above the cost-effectiveness threshold without herd-protection, the ICERs decreased below that threshold with inclusion of herd-protection (9 ICER per-QALYs and 8 ICER per-LYs analyses) (Table D in S1 File).

This phenomenon was observed only in analyses for more-developed countries. In all but one of the 29 ICER-analyses for less-developed countries, the target vaccination strategy was already below the cost-effectiveness threshold of X3GDP/capita even without inclusion of herd-protection. The ICER per-LY analysis for pneumococcal vaccine PCV7 for Brazil [55] was above the X3GDP/capita cost-effectiveness threshold and remained slightly above that threshold with herd-protection (the ICER per-DALY analysis for the same study was below the X3GDP/capita threshold without herd-protection).

### Subgroup analyses according to industry involvement, perspective (healthcare vs societal) and model used (dynamic vs static)

There was no difference in the number of ICER-outcome-analyses that crossed the cost-effectiveness threshold of $50,000 for more-developed countries and X3GDP/capita for less-developed countries (without vs with herd-protection) according to industry involvement, perspective analyzed or model used (among those that were above that threshold without herd-protection and were comparing a target vaccine against no vaccine). These thresholds were crossed in 50% (8/16) of those analyses with industry-involvement vs 41% (9/22) without industry-involvement (p = 0.58); in 44% (7/16) of those analyses with the societal perspective vs 45% (10/22) with the healthcare-perspective vs (p = 0.92) and in 54% (13/24) of those analyses with static-models vs 29% (4/14) with dynamic models (p = 0.13) (Table D in S1 File).

### Comparative analyses across ICER-metrics and vaccines

**Fig 3:**
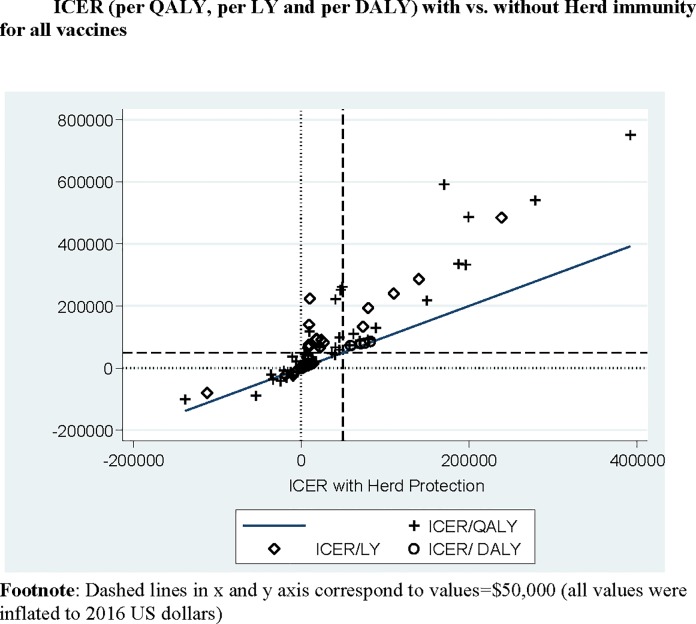
Scatterplot of ICERs (per-QALY gained, per-LY gained and per-DALY averted) with vs. without herd-protection across all four vaccines. Y-axis: ICERs without herd protection and X-axis: ICERs with herd-protection (values inflated to 2016 US dollars, [29]); Dashed lines in the horizontal and vertical axis correspond to $50,000 threshold without and with herd-protection respectively. (ICERs in the left upper quadrant indicate cases where ICERs were > $50,000 without herd-protection and crossed that threshold with Herd Protection).

The scatterplot of the three ICER-metrics, with vs without herd-protection, is shown in the in Fig 3. The ICER-differences varied significantly according to the metric used (p = 0.0006 by Kruskal-Wallis, for ICER-differences per-QALYs vs per-LYs vs per-DALYs) (Figure B-2 in S1 File). Moreover, in 59% (10/17) of ICER per-DALYs analyses the differences were <$100 [33, 48] (Table 2). These studies were in low-income countries and according to the World Bank, for low-income countries (with GDP/capita ≤ $735), interventions with ICERs per-DALY ≤$150 are considered attractive [71] (Table 2).

The differences in ICERs per-QALYs (without vs with herd-protection) across all four vaccines are depicted in the box-plot in Figure B-2 in S1 File and at the bar-plots in Fig 2 and Figure C in S1 File. (p = 0.0002 by Kruskal-Wallis). The respective differences for ICERs per-LYs and per DALYs across vaccines were not statistically significant (p = 0.79 and p = 0.37 respectively by Kruskal-Wallis) (Figure D-1 in S1 File and Figure E-1 in S1 File).

### Authors’ conclusions

The final authors’ conclusions appear in Table F in S1 File. The authors clearly recommended at least one target vaccination strategy in 69% (24/35) of studies and in another six studies considered that they could have been cost-effective under certain assumptions (herd-protection was considered among the key assumptions that would have changed the conclusions in 4 studies) (Table D in S1 File). Finally, 5 studies did not recommend the target vaccination strategies. Furthermore, the target vaccination strategy was clearly recommended in 83% (20/24) of industry-funded studies vs. 55% (6/11) of non-industry-funded studies (p = 0.07).

## Discussion

In this quantitative comparative analysis of the incremental-cost-effectiveness-ratios for four childhood vaccinations with vs without vaccine herd-protection, we showed that inclusion of herd-protection effects had a substantial impact in the estimated ICERs. In cases where the ICERs were above the cost-effectiveness threshold (of assumed societal-willingness-to-pay of $50,000 for more-developed countries or X3GDP/capita for less-developed countries) without herd-protection, inclusion of herd-protection led to crossing of that threshold in 45% of cases, making the target vaccination-strategy more attractive option. This impacted only CEAs for more-developed countries, as all but one of CEAs for less-developed countries had ICERs below the WHO-cost-effectiveness threshold even without herd-protection. We were not able to draw robust conclusions for true differences among those crossing the above thresholds with herd-protection, according to vaccine type, industry involvement status, perspective of analysis and CEA-model used due to the small numbers of analyses within individual subgroups. Among analyses that were not already cost saving without herd-protection, inclusion of herd-protection always gave more favorable results. Moreover, in a third of analyses that were already cost saving, the inclusion of herd-protection gave additionally more favorable cost-saving results.

The ICER-differences varied significantly according to the metric used, with the largest differences seen with the ICERs per-QALYs and per-LYs. The ICER-differences per-DALYs were small; however, even without herd-protection, the ICERs per-DALYs estimates were significantly smaller than ICERs per-QALYs or ICERs per-LYs estimates. In several studies, recommendation for the adoption of the target vaccination strategy depended on the inclusion of herd-protection effects in the calculations. Moreover, the ICER per-QALY differences varied according to vaccine, with the largest differences seen with pneumococcal and meningococcal vaccines. A possible explanation for that could be that the herd-protection effects considered for the pneumococcal and meningococcal vaccines were overall larger than those for the rotavirus and influenza vaccines; however the herd-protection assumptions considered varied significantly across studies even within the same vaccine group as shown in Table B in S1 File. Although no significant differences were detected across vaccines in the ICER-differences per LYs and per-DALYs, the data were more limited.

Empirical epidemiologic data on the size of vaccine-herd-protection effects in different countries were limited and most studies extrapolated herd-protection assumptions from other countries, used fixed herd-protection assumptions or applied modeling [72]. A prior systematic review by van de Vooren et al. [73] showed that among 10 European pneumococcal-conjugate vaccine CEA studies, only one study based herd-protection assumptions on national data, while most of the remaining studies used information for herd-protection and serotype substitution based on an American study. Although the approach of using assumptions rather than actual epidemiologic data for herd-protection might be appropriate for economic evaluations in settings where a vaccine is still being considered (2/10 studies), the majority of these studies were done in countries where the vaccines were already recommended [73]. Extrapolation of herd-protection effects from different countries should be cautiously done as differences in the dominant circulating strains, transmissibility of strains and other social factors (e.g. social mixing situations) as well as differences in the vaccination dosing schedules and vaccination coverage rates may impact the herd-protection effects [19]. Loo et al. [17] showed that indirect vaccine effects from pneumococcal-conjugate vaccines vary widely according to dosing schedule and endpoint studied (e.g. vaccine-serotype associated invasive pneumococcal disease, nasopharyngeal pneumococcal carriage, and pneumonia). This suggests that in order to be most useful, future CEA methodology must account for this complicated epidemiology.

It has been previously shown that most published economic-analyses (not limited to vaccines) reported favorable ICERs for the experimental interventions [74] and industry-sponsored economic-analyses in particular were more likely to report favorable ratios compared to non-industry sponsored CEA studies [74–76] In our sample of evaluated vaccine-CEAs the non-industry funded CEAs studies were less than a third of the total number of studies, to allow for detection of true between-group differences.

Our quantitative comparative analysis differs from prior systematic reviews of CEA studies for childhood vaccinations, as these prior reviews were mainly qualitative descriptive reviews. A detailed discussion of the differences between our analysis and these prior reviews is included in Text C in S1 file. In brief, in our quantitative comparative analysis with vs without herd protection: a) we applied very strict criteria for the ICER-analyses to be compared to optimize comparability of results (considering only ICER-analyses where all other parameters/assumptions, except for herd-protection, were the same); b) we calculated the size of the ICER-differences with vs without herd-protection across diverse childhood vaccines and for different ICER-metrics (per-QALYs, per-LYs and per-DALYs) to increase our power to detect true differences; c) we inflated all monetary-values to 2016 US dollars to increase comparability of results across studies and d) we explored factors that could explain the observed differences in the impact of herd-protection across studies, such as country setting, industry involvement status, CEA-perspective, vaccine type and CEA-model used.

Some study limitations should be acknowledged. We analyzed only the positive indirect vaccine effects from herd-protection. However, available data were very limited to allow for the performance of meaningful separate analyses for the impact of negative indirect vaccine effects, such as serotype substitution (either alone or in combination with herd-protection), in the ICER estimates. Only five pneumococcal vaccine CEA-studies included data with and without additional vaccine indirect effects. (We discussed those in detail in Text D in S1 File). We only analyzed economic analyses published in English; pertinent studies published in other languages [77, 78] might have been missed. We used benchmark cost-effectiveness-thresholds to assess the impact of including herd-protection in vaccine CEAs for more-developed countries. These thresholds are arbitrary [34, 79] but nevertheless are widely used to characterize interventions as cost-effective and worth adopting. Moreover, for less-developed low-income countries we applied the widely used WHO cost-effectiveness-thresholds of X3GDP/capita [39].

Overall, there is a need for continued surveillance and collection of robust empirical epidemiologic data on herd-protection positive vaccine effects and negative indirect vaccine effects e.g. from serotype substitution across diverse populations, countries, for different vaccination dosing schedules and vaccination coverage rates. Moreover, further methodological research is needed for the identification of the most efficient methods for incorporating herd-protection effects in economic analyses [19] There is progress towards that direction as international guidelines for the standardization of economic evaluations for vaccines have been recently developed by the European Vaccine Economics Community [80]. Dynamic models should be preferably used in those analyses, instead of static models, as they include the interaction between individuals and therefore account for indirect vaccine-effects [80]. The routine inclusion in vaccine economic analyses of the negative vaccine indirect effects, such as serotype substitution, in addition to the positive herd-protection effects, is necessary. Especially in the case of pneumococcal vaccination there is a need for more sophisticated models that count for carriage of different serotypes and not only for infection or illness. New approaches for the assessment of vaccine herd-protection, such as cluster-randomized trials that can assess vaccine-direct effects, herd-protection effects and negative indirect effects, e.g. from serotype replacement, even before the introduction of vaccines into public health programs, should be considered [81]. Moreover, pre-licensure assessment of vaccine herd-protection should not be used as a replacement for post-licensure assessments, as only post-licensure studies in diverse populations can provide an accurate estimate of vaccine’s herd-protection effects [81–84].

## Supporting information

S1 File (DOCX)
