# Supplementary material for: Impact of vaccine herd-protection effects in cost-effectiveness analyses of childhood vaccinations. A quantitative comparative analysis
Source: PLoS One. 2017 Mar 1;12(3):e0172414. doi: 10.1371/journal.pone.0172414 (PMC5332092; doi:10.1371/journal.pone.0172414)
Supplement: S1 File — (DOCX) [file pone.0172414.s001.docx]

­­­­S1 File

SUPPLEMENTARY INFORMATION

**Fig A:** Countries included in the 81 separate-analyses (across the 35 studies)

**Footnotes:**

Each CEA study could have included analyses for more than one countries.

Categorization of countries into more developed and less developed: Our definition of less-developed countries is consistent with the list of less developed countries of the International Monetary Fund. We considered more developed countries the following countries: United States, Canada, Australia, New Zealand, Israel, Japan, Singapore and Western European countries. All other countries, were considered as less developed.

**Abbreviations:** AFR: African region-GAVI eligible countries (Angola, Benin, Burkina Faso, Burundi, Cameroon, Central African Republic, Chad, Comoros, Congo, Congo DR, Cote D Ivoire, Eritrea, Ethiopia, Gambia, The Ghana, Guinea, Guinea-Bissau, Kenya, Lesotho, Liberia, Madagascar, Malawi, Mali, Mauritania, Mozambique, Niger, Nigeria, Rwanda, SaoTome & Principe, Senegal, Sierra Leone, Tanzania, Togo, Uganda, Zambia, Zimbabwe); AMR: Region of the Americas-GAVI eligible countries (Bolivia, Cuba, Guyana, Haiti, Honduras, Nicaragua), EMR: Eastern Mediterranean Region-GAVI eligible countries (Afganistan, Djibouti, Pakistan, Somalia, Sudan, Yemen), EUR: European region-GAVI eligible countries (Armenia, Azerbaijan, Georgia, Kyrgyzstan, Moldova, Tajikistan, Ukraine, Uzbekistan), GAVI: [Global Alliance for Vaccines and Immunization](http://www.acronymfinder.com/Global-Alliance-for-Vaccines-and-Immunization-(GAVI).html) ;SEAR: South East Asian Region-GAVIE eligible countries (Bangladesh, Bhutan, DPR Korea, India, Indonesia, Myanmar, Nepal, Sri Lanka, Timor-Leste); WPR: West Pacific region-GAVI eligible countries (Cambodia, Kiribati, Lao PDR, Mongolia, Papua New, Guinea, Solomon Islands, Vietnam).

**Fig B-1:** Differences in ICERs per-QALYs, per-LYs and per-DALYs (without-with herd protection) across all vaccines^a,b^

^
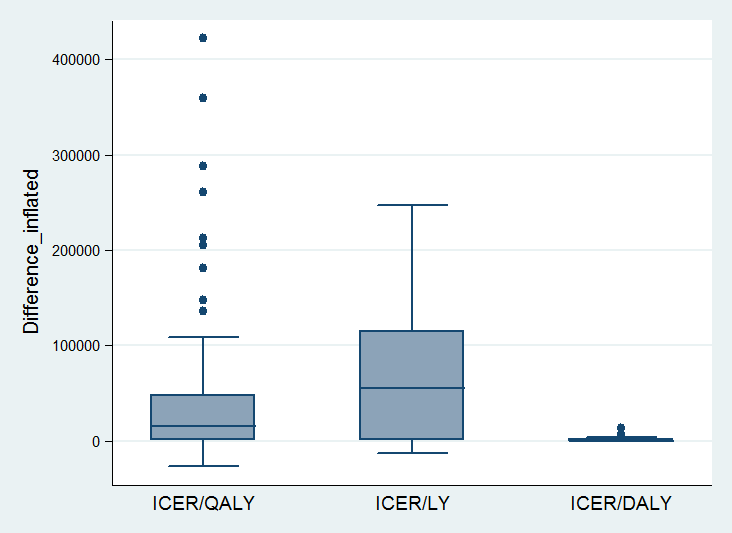
^

**Footnotes:**

1. Analyses are based on 55 ICER per-QALY analyses; 27 ICER per-LY analyses and 17 ICER per-DALY analyses across all four vaccines
2. CEAs studies might have not reported data for all 3 outcome metrics (ICER per-QALY, ­­ICER per-LY and ICER per-DALY) for the compared vaccines.
3. In all cases with negative difference between ICER without vs with herd protection, the respective ICER was already cost saving without herd protection, remained cost saving with herd protection; however the ratio was not incrementally more favorable compared to without herd protection

**Fig B-2:** Differences in ICERs per-QALYs (without-with Herd Protection) according to vaccine type (values are inflated to 2016 US dollars)^a,b^


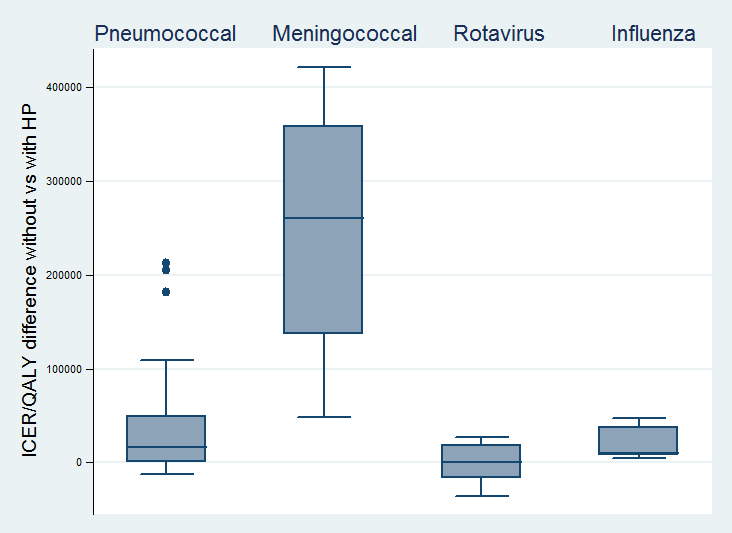


**Footnotes:**

a. Analyses are based on 28 pneumococcal, 7 meningococcal, 11 Rotavirus and 9 Influenza ICER per-QALYs analyses (ICER per-QALY differences with vs without Herd protection)

b. In all cases with negative difference between ICER per-QALY without vs with herd protection, the ICER per-QALY was already cost saving without herd protection, remained cost saving with herd protection; however the ratio was not incrementally more favorable compared to without herd protection.

**Fig C**: Differences in ICERs (per-QALYs) (without-with herd protection) per vaccine type (all values are inflated in 2016 US dollars)

**Footnotes:** In all cases with negative difference between ICERs per-QALYs without vs with herd protection, the ICERs per-QALYs were already cost saving without herd protection, remained cost saving with herd protection; however the ratio was not incrementally more favorable compared to without herd protection.

**Abbreviations:** P: pneumococcal conjugate vaccines, M: meningococcal conjugate vaccines; R: rotavirus vaccines; F: influenza vaccines

**Fig D-1**: Differences in ICERs (per-LYs) without vs with Herd Protection.(all values are inflated in 2016 US dollars)

**Abbreviations**: P: pneumococcal conjugate vaccines, M: meningococcal conjugate vaccines; R: rotavirus vaccines; F: influenza vaccines

**Fig D-2:** Analyses of ICERs (per-LYs) without vs with Herd Protection.

**Footnotes**: Grey bars: ICERs (per-LYs gained) without Herd Protection; Black bars: ICERs (per-LYs gained) with Herd Protection. (all values are inflated in 2016 US dollars)

**Abbreviations**: P= pneumococcal conjugate vaccines, M= meningococcal conjugate vaccines, R=rotavirus vaccines, F=influenza vaccines

**Fig E-1**: Differences in ICERs (per-DALYs) without vs with Herd Protection.(all values are inflated in 2016 US dollars)

**Abbreviations:** P= pneumococcal conjugate vaccines, M= meningococcal conjugate vaccines, R=rotavirus vaccines, F=influenza vaccines

**Fig E-2:** Analyses of ICERs (per-DALYs) without vs with Herd Protection (all values are inflated in 2016 US dollars)

**Footnotes:** Grey bars: ICERs per-DALYs averted without Herd Protection; Black bars: ICERs per-DALYs averted with Herd Protection

**Abbreviations**: P= pneumococcal conjugate vaccines, M= meningococcal conjugate vaccines, R=rotavirus vaccines, F=influenza vaccine

**Table A**: Characteristics of Included Studies, Analyses and ICER-Outcome Analyses

| **CEA studies** | **35 studies** |  |  |  | |
| --- | --- | --- | --- | --- | --- |
| Pneumococcal conjugate vaccines | 20 studies |  |  |  | |
| Meningococcal conjugate vaccines | 4 studies |  |  |  | |
| Rotavirus vaccines | 8 studies |  |  |  | |
| Influenza vaccines | 3 studies |  |  |  | |
| **Analyses (for different compared vaccines; perspectives [healthcare or societal] or countries)** | **81 analyses** |  |  |  | |
| Pneumococcal conjugate vaccines | 37 analyses |  |  |  | |
| Meningococcal conjugate vaccines | 13 analyses |  |  |  | |
| Rotavirus vaccines | 22 analyses |  |  |  | |
| Influenza vaccines | 9 analyses |  |  |  | |
| **Industry Involvement** | **24/35 (69%) of the studies** |  |  |  | |
|  | 64/99 (64%) of the ICER-outcome analyses |  |  |  | |
| **ICER-Outcome Analyses** | **99 ICER-analyses** |  |  |  | |
| ICERs per-QALYs | 55 |  |  |  | |
| ICERs per-LYs | 27 |  |  |  | |
| ICERs per-DALYs | 17 |  |  |  | |
| **ICER-analyses per Vaccine Type (N=99)** |  |  |  |  | |
| Pneumococcal conjugate vaccines | 53 |  |  |  | |
| Meningococcal conjugate vaccines | 14 |  |  |  | |
| Rotavirus vaccines | 22 |  |  |  | |
| Influenza vaccines | 10 |  |  |  | |
|  |  |  |  |  | |
|  | **Pneumococcal** | **Meningococcal** | **Rotavirus** | **Influenza** | **Total** |
| **Total ICER-outcome Analyses (N=99)** | **53** | **14** | **22** | **10** | **99** |
| ICERs (per-QALYs) | 28 | 7 | 11 | 9 | 55 |
| ICERs (per-LYs) | 19 | 7 | 0 | 1 | 27 |
| ICERs (per-DALYs) | 6 | 0 | 11 | 0 | 17 |
| **Models (N=35 studies)** |  |  |  |  |  |
| Static Cohort/Population models | 29 studies |  |  |  |  |
|  | Herd protection was included in the Base Case Scenario in 6 of these studies |  |  |  |  |
| Dynamic transmission models | 6 studies |  |  |  |  |
| **Compared Strategies (N=99 ICER-analyses)** |  |  | | | |
| Target Vaccination strategy vs No vaccine | 79 (80%) |  | | | |
| Target Vaccination strategy vs Another vaccination strategy | 20 (20%) |  | | | |
| **ICER-analyses per Country Setting (N=99 ICER-analyses)** |  |  | | | |
| CEAs for More Developed Countries | 70/99 (71%) |  | | | |
| ICERs (per-QALYs) | 47 |  | | | |
| ICERs (per-LYs) | 19 |  | | | |
| ICERs (per-DALYs) | 4 |  | | | |
| CEAs for Less Developed Countries | 29/99 (29%) |  | | | |
| ICERs (per-QALYs) | 8 |  | | | |
| ICERs (per-LYs) | 8 |  | | | |
| ICERs (per-DALYs) | 13 |  | | | |
| **ICER-analyses per Perspective (N=99 ICER-analyses)** |  |  | | | |
| Health care perspective | 69/99 (70%) |  | | | |
| Societal Perspective | 30/99 (30%) |  | | | |
| **Cost Saving analyses-Without Herd Protection** | **16/99 (16%)** | | | | |
| ICERs (per-QALYs) | 12 |  | | | |
| ICERs (per-LYs) | 4 |  | | | |
| ICERs (per-DALYs) | 0 |  | | | |
| Subgroup analysis  -according to Country-setting (N=16) | 13 for more developed countries | 3 for less developed countries | | | |
| Subgroup analysis  -according to Vaccine type (N=16) | 8 P; 6 R; 2 F |  | | | |
| Subgroup analysis  -according to CEA-model (N=16) | 11 with Static model | 5 with Dynamic model | | | |
| **Below the cost-effectiveness threshold**  **($50000 for more developed countries orX3GDP/capita for less developed countries)**  **even without Herd Protection**  **(among those 79 ICER-analyses comparing a target vaccination strategy vs no vaccine)** | **41 ICER-analyses** | | | | |
| Subgroup analysis  -according to Country-setting (N=41) | 13 for more developed countries | 28 for less developed countries | | | |
| Subgroup analysis  -according to Vaccine type (N=41) | 24 P, 2 M; 9 R; 6 F |  | | | |
| Subgroup analysis  -according to CEA-model (N=41) | 33 with Static model | 8 with Dynamic model | | | |

**Table B:** Herd Protection Effect Assumptions

| Vaccine (Country) | Author | | Year | Direct reference for herd immunity assumptions | Description of herd immunity assumptions |
| --- | --- | --- | --- | --- | --- |
| *Pneumococcal* | | | | | |
| PCV7-Sweden | Bergman, et al. | 2008 | | Wisloff, et al. *Cost-effectiveness of adding 7-valent pneumococcal conjugate (PCV-7) vaccine to the Norwegian childhood vaccination program.* Vaccine 2006; 24:5690-9. | Accounted for herd immunity in adults only. Used age-specific estimated reduction in invasive pneumococcal disease. [9.3% (18-40 yrs); 13.8% (40-65 yrs); 18.7% (>65 yrs)] |
| PCV13, PCV7-Switzerland | Blank, et al. | 2012 | | 1) Strutton et al. *Cost-effectiveness of 13-valent pneumococcal conjugate vaccine: Germany, Greece, and The Netherlands*. Journal of Infection 2012; 64:54-67.  2) Moore M. The Center’s for Disease Control and Prevention Active Bacterial Core Surveillance (ABCs) program; 2009  3) CDC, Case definition invasive pneumococcal disease, Streptococcus pneumonia, invasive disease, Laboratory criteria for diagnosis. 2010.  4) Whitney CG, et al. *Decline in invasive pneumococcal disease after the introduction of protein-polysaccharide conjugate vaccine.* NEJM 2003; 348:1737-46. | Used Strutton et al. data for PCV-7 herd immunity. Based indirect effectiveness of PCV-13 on that of PCV7 and its proportional serotype coverage after dividing additional age-specific serotype adjustment by two (conservative approach.) Applied herd effect to all age groups, had different estimates for invasive pneumococcal disease, hospitalized pneumonia, and complex otitis media. “After 7 years of initial vaccination, the indirect effect reached a cumulative steady state of 100%.” (Indirect effect for IPD: 48% for <12 mo; 56% for 12-23 mo; 43% for 24-35 mo; 43% for 36-47 mo; 41% for 48-59 mo; 37% for 5-17 yrs; 32% for 18-34 yrs; 30% for 35-49 yrs; 27% for 50-64 yrs; and 14% for +65 yrs) |
| PCV13, PCV10, PCV7-Canada | Chuck, et al. | 2010 | | 1) Morrow A, et al. *The burden of pneumococcal disease in the Canadian population before routine use of the seven-valent pneumococcal conjugate vaccine.* Can J Infect Dis Med Microbiol 2007; 18(2): 121-7.  2) Tyrrell GJ, et al. S*erotypes and antimicrobial susceptibilities of invasive Streptococcus pneumonia pre- and post-seven valent pneumococcal conjugate vaccine introduction in Alberta, Canada, 2000-2006.* Vaccine 2009; 27:3553-60. | Indirect protection for invasive and non-invasive pneumococcal disease estimated by comparing the incidence rates of PCV7 serotypes before and after the introduction of PCV7 in Canada (2000-2002 versus 2003-2008) in individuals ≥2 years old [decrease by 60% in 2-4 yrs; 28% in 5-9 yrs; 31% in 10-14 yrs; 6% in 15-19 yrs; 26% in 20-39 yrs; 37% in 40-64 yrs; 50% in 65+ yrs] |
| PCV13-Spain | Díez-Domingo, et al. | 2011 | | 1) Dirección General de Salud Pública Área de Epidemiología [Homepage]. Análisis de Vigilancia Epidemiológica (AVE). Valencia; 2010.  2) Dirección General de Salud Pública. Generalitat Valenciana [Homepage]. Conjunto Minimo de Bases de Datos de la Comunidad Valenciana. Valencia; 2010.  3) Rozenbaum MH, et al. *Observed differences in invasive pneumococcal disease epidemiology after routine infant vaccination.* Expert Rev Vaccines 2011; 10:187-99. | Indirect protection for adults estimated from 0 – 20% (0%, 5%, 10%, 15%, 20% Herd effect) decrease of the incidence of severe diseases in adults based upon references. Base case included 5% which authors considered mid range of data published in Europe based upon Rozenbaum et al. |
| PCV13, PCV10-Canada | Earnshaw, et al. | 2012 | | 1) Ray GT, et al. *Cost-effectiveness of pneumococcal conjugate vaccine: evidence from the first five years of use in the United States incorporating herd effects.* Pediatr Infect Dis J 2006,25:6.  2) Fireman B, et al. *Impact of the pneumococcal conjugate vaccine on otitis media.* Pediatr Infect Dis J 2003, 22:10-16.  3) Zhou F, et al. *Health care utilization for pneumonia in young children after routine pneumococcal conjugate vaccine use in the United States.* Arch Pediatr Adolesc Med 2007, 161:1162-1168.  4) Hansen J, et al. *Effectiveness of heptavalent pneumococcal conjugate vaccine in children younger than 5 years of age for prevention of pneumonia: updated analysis using World health Organization standardized interpretation of chest radiographs.* Pediatr Infect Dis J 2006, 25:779-781. | Indirect effects for PCV7 based upon references. Indirect effects for PCV13 and PCV10 were based upon PCV7 data. Considered indirect protection against invasive pneumococcal disease (e.g. 68% reduction for 0-2 yrs; 68% for 3-4 yrs; 39% for 5-17 yrs; 47% for 18-64 yrs and 36% for 64+ yrs) , pneumonia for all age groups, and acute otitis media for unvaccinated children <2 years of age. |
| PCV7-Argentina | Giglio, et al. | 2010 | | 1) CDC. *Direct and indirect effects of routine vaccination of children with 7-valent pneumococcal conjugate vaccine on incidence of invasive pneumococcal disease – United States 1998 – 2003.* MMWR Morb Mortal Wkly Rep 2005; 54 (September (36)): 893-7.  2) Hsu, et al. *Effect of pneumococcal conjugate vaccine on pneumococcal meningitis.* NEJM 2009; 360 (January (3)): 244-56. | " If we assume the indirect (herd) effect as seen in the US, in which for every IPD case prevented in a vaccinated cohort there are 2.2 IPD cases prevented in unvaccinated leading to a 65% reduction of the incidence of IPD in people over 65 years" |
| PCV10-Peru | Gomez, et al. | 2013 | | 1) Centers for Disease Control and Prevention (CDC): Direct and indirect effects of routine vaccination of children with 7-valent pneumococcal conjugate vaccine on incidence of invasive pneumococcal disease - United States, 1998–2003. MMWR Morb Mortal Wkly Rep 2005, 54(36):893–897.  2) Centers for Disease Control and Prevention (CDC): Invasive pneumococcal disease in children 5 years after conjugate vaccine introduction - eight states, 1998–2005. MMWR Morb Mortal Wkly Rep 2008, 57(6):144–148. | “Herd effect (evaluated as a fixed incidence reduction of IPD of 15.4% and 29.0% among children <5 years and ≥5 years, respectively, based on the experience reported by CDC in the USA.” |
| PCV7-Japan | Hoshi, et al. | 2012 | | Leino T, et al. *Indirect protection obtained by Haemophilus influenza type b vaccination: analysis in a structured population model.* Epidemiol Infect 2004; 132:959-66. | Indirect protection for <5 years old only. For 65% vaccine uptake rate, estimated indirect protection from Leino et al. “Reduction of transition probabilities of invasive pneumococcal disease: 64.9%, 35.9%, 19.4%, 8.9%, 26.8% (for 1st to 5th year); of hospitalized pneumonia: 0.0%, 13.1%, 15.3%, 19.2%, 21.3% (from 1st to 5th year); of serotype AOM 25.3%,29.7%, 31.5%, 34.7%, 36.4% (from 1st to 5th year after vaccination initiation)” |
| PCV7-Netherlands | Hubben et al. | 2007 | | Whitney CG, et al. *Decline in invasive pneumococcal disease after the introduction of protein-polysaccharide conjugate vaccine.* NEJM 2003; 348:1737-46. | Incorporated indirect protection using estimated decline in invasive pneumococcal disease in adults (20 years and older) from Whitney et al. Assumed indirect effects lasted 1 year. [20–39 years decline by 32% (95% CI, 23–39); in 40–64 years by 8% (1–15); in ≥ 65 years by18%] |
| PCV13, PCV9/PCV10, PCV7-Gambia | Kim et al | 2010 | | 1) Whitney CG, et al. *Decline in invasive pneumococcal disease after the introduction of protein-polysaccharide conjugate vaccine.* NEJM 2003; 348:1737-46.  2)Adegbola RA, et al. *Serotype and antimicrobial susceptibility patterns in isolates of Streptococcus pneumonia causing invasive disease in The Gambia 1996-2003.* Tropical Medicine and International Health 20006, 11(7):1128-1135. | Assumed reduction in invasive pneumococcal disease incidence in unvaccinated individuals per Whitney et al.  Varied incidence of diseases among unvaccinated individuals aged 5-19 years. Adjusted level of extrapolated herd immunity using estimated ratios of the age group-specific serotype coverage in The Gambia versus the US [assumed 32%, 32%, 8%, and 18% decrease in incidence of primary endpoint pneumonia and pneumococcal meningitis and sepsis for unvaccinated individuals aged 4-19 years, 20-39 years, 40-64 years, and >65 years, respectively. |
| PCV10-Argentina, Peru, Chile, Colombia, Brazil, Mexico | Marti et al. | 2013 | | 1) Ardanuy C, Tubau F, Pallares R, Calatayud L, Dominguez MA, Rolo D, et al: Epidemiology of invasive pneumococcal disease among adult patients in Barcelona before and after pediatric 7-valent pneumococcal conjugate vaccine introduction, 1997-2007. Clin Infect Dis 2009, 48(1):57–64. Jan 1.  2) Hanna JN, Humphreys JL, Murphy DM: Invasive pneumococcal disease in Indigenous people in north Queensland: an update, 2005-2007. Med J Aust 2008, 189(1):43–46. Jul 7.  3) LA Hicks HL, Flannery B, Hadler JL, et al: Incidence of pneumococcal disease due to non-pneumococcal conjugate vaccine (PCV7) serotypes in the United States during the era of widespread PCV7 vaccination, 1998-2004. J Infect Dis 2007, 196(9):1346–1354.  4) Kellner JD, Church DL, MacDonald J, Tyrrell GJ, Scheifele D: Progress in the prevention of pneumococcal infection. CMAJ 2005, 173(10):1149–1151. [Research Support, Non-U.S. Gov’t]. 2005 Nov 8. | “As the herd immunity varies across countries with different effects, a secondary analysis estimated indirect effects of vaccination on the population (herd immunity and serotype replacement).”  A net reduction of 15.4% was assumed in children under five and a net reduction of 29% was assumed from five years old until death. |
| PCV7-UK | McIntosh, et al. | 2005 | | Whitney CG, et al. *Decline in invasive pneumococcal disease after the introduction of protein-polysaccharide conjugate vaccine.* NEJM 2003; 348:1737-46. | Assumed reduction in invasive pneumococcal disease incidence in unvaccinated individuals per Whitney et al. Also applied to 13.4% of unspecified hospital-treated pneumonia. Applied the lower end of the 95% confidence intervals of the published effects: 23% for 20 to 39 years, 1% for 40–64 years and 11% for 65+ years. |
| PCV7-England-Wales | Melegaro, et al. | 2004 | | Whitney CG, et al. *Decline in invasive pneumococcal disease after the introduction of protein-polysaccharide conjugate vaccine.* NEJM 2003; 348:1737-46. | Assumed reduction in invasive pneumococcal disease incidence in unvaccinated individuals per Whitney et al. Assumed protection for 1 year. They estimated the following reduction in IPD incidence among unvaccinated individuals: 32% (95% CI:  23–39%) in the 20–39 years old, 8% (95% CI: 1–15%) in  the 40–64 years and 18% (95% CI: 11–24%) in the 65+ 1 year after the introduction of infant vaccination. |
| PCV13, PCV10, PCV7-Australia | Newall, et al. | 2011 | | 1) Roche et al. *Invasive pneumococcal disease in Australia, 2006.* Commun Dis Intell 2008; 32(1):18-30.  2) Australian Department of Health and Ageing. National Notifiable Diseases Surveillance System. | 21% reduction in invasive pneumococcal disease due to PCV-7 based upon reference (for 5-64 years). Taking into account additional IPD serotype coverage, the authors estimated an incremental herd protection of 2.6% for PHiD-CV and 11.7% for PCV-13 based upon national data. Applied to all unvaccinated individuals 2 years after the introduction of the new vaccine strategy |
| PCV7-USA | Ray, et al. | 2006 | | Centers for Disease Control and Prevention’s Active Bacterial Core Surveillance (ABCs) program; Whitney (NEJM 2003) | Assumed herd effects for the first 5 years after introduction of PCV.  Herd effects for unvaccinated differed by age: <5 years old - inferred from the difference between the actual in reduction of IPD cases from ABCs data and estimated reduction from vaccination to indirect effect. > 5 years old -- based upon observed rates of IPD in the post-vaccine years compared with average rates from 1997 – 1999. (68% for <5 yrs; 38% for 5-15 yrs; 47% for 15-45 yrs; 20% for 45-65 yrs; 36% for 65+ yrs) |
| PCV13, PCV7-USA | Rubin, et al. | 2010 | | 1) Robinson KA, Baughman W, Rothrock G, et al. Epidemiology of invasive Streptococcus pneumoniae infections in the United States, 1995–1998: Opportunities for prevention in the conjugate vaccine era. JAMA. 2001; 285:1729 –1735.  2) Annual estimates of the population by sex and five-year age groups for the United States: April 1, 2000 to July 1, 2004 (NC-EST2004-01).  3) U.S. Dept. of Health and Human Services, National Center for Health Statistics. National Ambulatory Medical Care Survey, 2006.  4) U.S. Dept. of Health and Human Services, National Center for Health Statistics. National Hospital Ambulatory Medical Care Survey, 2006.10  5) Zhou F, et al. Health care utilization for pneumonia in young children after routine pneumococcal conjugate vaccine use in the United States. Arch Pediatr Adolesc Med 2007, 161:1162-1168.  6)Grijalva CG, Nuorti JP, Arbogast PG, Martin SW, Edwards KM, Griffin MR.Decline in pneumonia admissions after routine childhood immunisation with pneumococcal conjugate vaccine in the USA: a time-series analysis. Lancet 2007; 369(9568): 1179–86. | Accounted for herd effect for IPD for all ages, all-cause inpatient and outpatient pneumonia for >5 years old, and otitis media <2 years old. Used references and immunogenicity data to estimate projected herd effect of PCV13 and applied these projects to 2007 incidence data. Accounted for 7 years of effect ( Reduction of IPD (per ABC/CDC data ) <1 yr=44%; 1-2 yrs=22%; 2-5 yrs=39%; 5-17 years=35%; 18-34 yrs=37%; 35-49 yrs= 32%; 50-64 yrs= 25%; +65 yrs= 27%)  ­­­­ |
| PCV13, PCV10, PCV7-Singapore | Tyo, et al. | 2011 | | 1) Ray GT, et al. Cost-effectiveness of pneumococcal conjugate vaccine: evidence from the first 5 years of use in the United States incorporating herd effects. Pediatr Infect Dis J 2006; 25(June (6)): 494–501.  2) Butler JR, McIntyre P, MacIntyre CR, Gilmour R, Howarth AL, Sander B. The cost-effectiveness of pneumococcal conjugate vaccination in Australia. Vaccine 2004; 22(March (9–10)): 1138–49. | All-cause pneumonia and IPD for non-vaccinated individuals >5 years old. Set herd effects in the unvaccinated population to 20% of direct effects to account for varied reports of herd effect in European populations compared to the American experience. |
| PCV13, PCV10-Argentina | Uruena, et al. | 2011 | | none | HI: 5% and 10%. Herd effect limited to < 5 years old. Varied herd effect between 0 – 10 % (% increase in health benefits among children <5 years) |
| PCV7-Brazil | Vespa, et al. | 2009 | | Sistema de Informações Ambulatoriais (SIA/SUS), DATASUS 2006. Available from: http://www.datasus.gov.br/. Accessed 14 March 2007. | HI: 0% to 49%. Estimates of vaccine protection for non-vaccinated populations (herd immunity) were based on published 11­­­­­estimates of invasive pneumococcal disease incidence and case fatality rates among U.S. adults. We then applied the proportion reductions in invasive pneumococcal disease seen among U.S. adults to the Brazilian incidence estimates in order to calculate cases and deaths averted due to herd immunity among older children, adolescents and adults. DALYs averted were based on the estimated median age at time of disease. |
| PCV7-Norway | Wisløff, et al. | 2006 | | 1) Whitney CG, et al. *Decline in invasive pneumococcal disease after the introduction of protein-polysaccharide conjugate vaccine.* NEJM 2003; 348:1737-46.  2) Pedersen MK, et al. Systemic pneumococcal disease in Norway 1995–2001:capsular serotypes and antimicrobial resistance. Epidemiol Infect 2004; 132(2):167–75. | Adapted reduction in IPD reported by Whitney, et al. to for pneumococcal serotypes infecting adults in Norway. (Reductions: 8.9% (for 20-39 yrs group), 12.9% (for 40-64 yrs group) and 22.9% (for the oldest group) respectively. |
| ***Meningococcal*** | | | | | |
| Men B-UK  (MenB at 1,4,6,and 12 mo) | Christensen, et al. | | 2013 | 1) Edmunds WJ, Medley GF, Nokes DJ. Evaluating the cost-effectiveness of vaccination programmes: a dynamic perspective. Stat Med 1999;18: 3263–82.  2) Christensen H, May M, Bowen L, et al. Meningococcal carriage by age: a systematic review and meta-analysis. Lancet Infect Dis 2010;10: 853–61.  3) Mossong J, Hens N, Jit M, et al. Social contacts and mixing patterns relevant to the spread of infectious diseases. PLoS Med 2008;5:e74. | “We therefore used two types of model, including a transmission dynamic model, in order to appropriately capture potential herd immunity effects. Unlike previous meningococcal models, our carriage estimates were drawn from a recent systematic review and meta-analysis and we assessed the impact of assuming different mixing patterns in the population using simple preferential mixing and mixing based on self-reported contacts.” |
| Meningococcal polysaccharide vaccines (ACYW135 or AC) Canada  (mass immunization of 6 mo-20 yrs during outbreak) | De Wals, et al. | | 2002 | De Wals P, et al. *Impact of a mass immunization campaign against serogroup C meningococcus in the province of Quebec, Canada.* Bull WHO 1996;74:407-11  De Wals, et al. *Five-year study of the effectiveness of polysaccharide vaccine against serogroup C meningococcal disease*. JAMA 2001; 285:177-81. | Estimated the number of cases prevented based upon reference. Restricted herd immunity to age group targeted by campaign (6 months-20 years) and ≤ 1 year duration. (Among non-vaccinated persons aged 6 months to 20 years, the incidence increased from 31.5 per million in 1990, to 49.7 in 1991 and 47.3 in 1992. During the first year after the mass immunization campaign, the incidence decreased to 30.1 per million in the non-vaccinated fraction of the target population, indicating a slowing down in the transmission of the pathogen in this cohort (36% reduction) |
| Men ACYW135, MenC-Netherlands | Hepkema, et al. | | 2013 | Rozenbaum MH, Sanders EA, van Hoek AJ, Jansen AG, van der Ende A, et al. (2010) Cost effectiveness of pneumococcal vaccination among Dutch infants: economic analysis of the seven valent pneumococcal conjugated vaccine and forecast for the 10 valent and 13 valent vaccines. BMJ 304: c2509. | “Herd immunity accounted for only the first year of vaccination”  “Herd-immunity was incorporated in the model in a similar way as Rozenbaum et al. did.” The magnitude of the herd-immunity was obtained by comparing the serogroup C incidence of 2001 with the average serogroup C incidence of 2007–2011. The incidence declined with 92% in both children between 0 and 1 years of age and in persons aged 27 years and over. As both groups were not protected by direct vaccination during both periods, this figure was used as the magnitude of herd-immunity induced by the vaccination. The decline in other age-groups, assuming a duration of protection of 5 years for vaccination at 14 months and of 25 years for vaccination at 12 years, was in accordance with a herd immunity effect of 95% in age-categories that were also protected by direct vaccination and of 92% in all other age-groups. |
| MenC-England&  Wales  (MCV-C) | Trotter, et al. | | 2006 | Maiden MCJ, et al on behalf of the UK Meningococcal Carriage Group. *Carriage of serogroup C meningococci one year after meningococcal C conjugate polysaccharide vaccination.* Lancet. 2002; 359:1829-30. | Herd immunity was the result of 67% protection against carriage acquisition of vaccinated individuals. Risk of carriage is dependent on age (highest for adolescents.) |
|  |  |  |  |  |  |
| ***Rotavirus*** | | | | | |
| R- All GAVI, Central/South America, EU, Africa, Eastern Mediternean, SE Asia, West Pacific | Atherlly, et al. | | 2012 | none | Assumed that “unvaccinated children would receive half of the level of protection as vaccinated children, times the proportion of children vaccinated.“ So at 50% coverage and 60% efficacy in vaccinated children, unvaccinated would receive 15% protection, while at 95% coverage, unvaccinated children would receive 28.5% protection. These simplified assumptions are intended to provide a preliminary estimate of the potential impact. |
| RotaTeq-England & Wales | Atkins, et al. | | 2012 | 1) Atkins, et al. *Impact of rotavirus vaccination on epidemiological dynamics on England and Wales.* Vaccine 2012; 30:552-64*.*  2) Pitzer V, et al. *Direct and Indirect Effects of Rotavirus Vaccination: Comparing Predictions from Transmission Dynamic Models.* PLoS ONE 2012; 7(8): e423. | Model implicitly takes into account herd immunity by including all individuals and accounting for onward transmission. In particular, our models predict that, after transient dynamics have subsided, indirect effects for children under five years alone account for an average 29% (vaccine waning) or 35% (no vaccine waning) reduction of any RVGE incidence at 95% vaccine coverage. |
| Rotarix-Turkey | Bakir, et al. | | 2013 | Standaert, et al. *Impact of Rotavirus Vaccination on Hospitalisations in Belgium: Comparing Model Predictions with Observed Data.* PLOSOne 2013;8(1):e53864 | Fixed herd effect of 10% improvement in vaccine efficacy applied only to infants <3 months of age |
| R-Netherlands | Bruijning-Verhagen, et al. | | 2013 | 1) Vesikari T, Itzler R, Karvonen A, Korhonen T, Van DP, Behre U, Bona G, Gothefors L, Heaton PM, Dallas M, Goveia MG: RotaTeq, a pentavalent rotavirus vaccine: efficacy and safety among infants in Europe. Vaccine 2009, 28:345–351.  2) Vesikari T, Karvonen A, Ferrante SA, Ciarlet M: Efficacy of the pentavalent rotavirus vaccine, RotaTeq(R), in Finnish infants up to 3 years of age: the Finnish Extension Study. Eur J Pediatr 2010, 169:1379–1386.  3) Vesikari T, Dennehy P, Matson D, Itzler R, Dallas M, Goveia M, DiNubile M, Heaton P, Lawrence J, Ciarlet M: Efficacy of Rotateq®, the pentavalent rotavirus vaccine, between doses: potential benefits of early protection [abstract]. Arch Dis Child 2008, 93:pw 70. | “Indirect vaccination effects (herd-immunity) among unvaccinated children were considered as part of the sensitivity analysis.” (30% [0% to 46%]; Observed effects among unvaccinated individuals ranged from 0 to 72% with substantial differences between consecutive years and effects declining with increasing age) |
| Rotarix, RotaTeq-Belgium, England & Wales, Finland, France, Netherlands | Jit, et al. | | 2009 | Van Effelterre T, et al. *Potential impact of high vaccine coverage against rotavirus diarrhea in Belgium.* In: Vaccine Congress. Amsterdam: Elsevier B.B.; 2007. | An age-specific relative increase in cases averted of RVGE in children under 5 yrs old was incorporated (relative increase: “79%, 32%, 16%, 11% and 8% for children in their 1st, 2nd, 3d, 4th and 5th year of life respectively.") Not adjusted for vaccine coverage rates or by country. |
| Rotarix, RotaTeq-Netherlands | Mangen, , et al. | | 2010 | none | Evaluated 2 scenarios: ”10% and 50% decrease of annual rotavirus infections in the non-vaccinated population” |
| R-Netherlands | Rozenbaum , et al. | | 2011 | none | Assumed herd protection would be as effective as completing all doses of vaccine. Applied to unvaccinated children and those who had not completed vaccination series. Herd protection was assumed for those not yet (fully) protected by the vaccine (either too young to be vaccinated or those who had not yet received the complete set of doses) and non-vaccinated children (5% of a birth cohort for the Dutch situation), assuming protection would be as effective as the vaccination would be after completing all doses. |
| RotaTeq-Netherlands | Tu, et al. | | 2013 | 1) Rozenbaum MH, et al. *Cost-effectiveness of rotavirus vaccination in the Netherlands; the results of a consensus model.* BMC Public Health 2011, 11:462.  2) Lopman BA, et al. *Infant rotavirus vaccination may provide indirect protection to older children and adults in the United States.* JID 2011, 2014:980-986. | For children <5 years old, assumed herd protection would be as effective as completing all doses of vaccine. Applied to unvaccinated children and those who had not completed vaccination series. For individuals 5-24 years old, based herd immunity on reduced rate of rotavirus gastroenteritis hospitalizations (relative risk 0.29 5-14 years old, 0.35 15-24 years old) |
| *Influenza* | | | | | |
| F-USA | Clements, et al. | | 2011 | Weycker D, Edelsberg J, Halloran ME, et al. Population-wide benefits of routine vaccination of children against influenza. Vaccine 2005; 23:1284.93. | Derived probability of influenza-like illness by age group based upon reference. (direct immunity; indirect + direct immunity) = 0-4 years (0.40- 0.24=16%); 5-17 yrs (0.2- 0.12=8%); 18-64 yrs (0.2- 0.16=4%); 65yrs+ (0.32- 0.26=5% |
| F-TIV-Australia | Newall, et al. | | 2013 | Newall AT, Dehollain JP, Wood J. Under-explored assumptions in influenza vaccination models: implications for the universal vaccination of children. Vaccine. 2012;30(39):5776–81. | “We adapted a previously constructed age-stratified Susceptible Exposed Infectious Recovered (SEIR) model (described in [27]) to estimate the underlying transmission of influenza under alternative vaccination scenarios. The model was able to estimate the herd protection conferred to the population through vaccination.” |
| F-TIV, LAIV-England & Wales | Pitman, et al. | | 2013 | Pitman RJ, White LJ, Sculpher M. Estimating the clinical impact of introducing paediatric influenza vaccination in England and Wales. Vaccine 2012. 30(6):1208-24. | “…In order to investigate the contribution made by indirect protection, arising from herd immunity, in those not targeted for vaccination, the cost-effectiveness acceptability frontier was recalculated using costs and QALYs derived exclusively from the age groups targeted for paediatric vaccination. …” (Data for Direct effects only, provided in Appendix Table A5) |
|  |  |  |  |  |  |

**Footnotes**: Citations for included studies = Reference 35-69 in main manuscript

*In the following fours studies, analyses for a range of herd protection assumptions were reported and we had decided a priori to keep the analysis for a conservative herd protection rate closest to 15%: Diez [38] (PCV13-Spain) : 0%, 5%, 10%, 20%; Tyo [51](PCV13, PCV10, PCV7 Singapore): 0%, 5%, 10%, 20%, 40%, 60%, 80%, 100% (base case had considered 20% herd protection and additional sensitivity analyses 0% and 40%); Uruena [52](PCV13, PCV10, Argentina): 5%, 10% and Atherly [59](Rotavirus, GAVI countries): 15% (for 50% vaccine coverage and 60% efficacy) and 28.5% (for 95% vaccine coverage)*

**Table C:** Methodologies used in CEA studies (The classification of models was according to Ultsch B et al. Methods for Health Economic Evaluation of Vaccines and Immunization Decision Frameworks: A Consensus Framework from a European Vaccine Economics Community; Pharmacoeconomics 2015)

| Vaccine (Country) | Author | Reference | Model used* | Target Cohort/Population | Vaccination Coverage assumptions | Monetary unit |
| --- | --- | --- | --- | --- | --- | --- |
| PCV7-Sweden | Bergman A, Hjelmgren J, Ortqvist A, Wisloff T, Kristiansen IS, Hogberg LD, Persson KM-S, Persson U | Scand J Infect Dis. 2008; 40:721-9. | Static Cohort model (Markov model, [base case: without HP; sensitivity analyses with HP]) | Hypothetical Swedish birth cohort of 95,000 infants (follow up over life time) | 100% | 2006 euro |
| PCV13, PC7-Switzerland | Blank, P. R., & Szucs, T. D. | Vaccine 2012; 30: 4267–4275 | Static Cohort Decision-analytic model (base case: without HP; sensitivity analyses: with HP) | Hypothetical birth cohort of 73,019 individuals in Switzerland with distinction made between different age groups (10 year time horizon) | 83% | 2012 euro |
| PCV13, PCV10, PCV7-Canada | Chuck Anderson W, PhilipJacobs, GregoryTyrrell, JamesD.Kellnerd, | Vaccine. 2010; 28: 5485-90. | Static Population model (base case: without HP; additional analyses with HP reported); steady state simulation model | Entire Alberta, Canada population in 2006 | 84% (<2 years) | 2008 Canadian dollar |
| PCV13-Spain | Díez-Domingo J, Ridao-López M, Gutiérrez-Gimeno MV, et al. | Vaccine 2011; 29 :9640–9648 | Cohort model (Decision tree; *[base case with 5% HP*; but additional analyses without HP and 10% and 20% HP were also reported]) | 10 hypothetical birth cohorts of 543,971 children in Spain; (follow up over life time) | 95% | 2009 euro |
| PCV13, PCV10-Canada | Earnshaw, S. R., McDade, C. L., Zanotti, G., Farkouh, R. A., & Strutton, D. | BMC Infectious Diseases 2012, 12:101 | Static Population model (Decision-analytic model [base case: without HP; additional analyses: with HP) | Population of 34,108,000 individuals in Canada; (follow up over life time during which they are at risk for IPD, PNE, or AOM based on their vaccination status; individuals entered the model either vaccinated or not vaccinated, depending upon their ages and vaccination uptake) | 91% | 2010 Canadian dollar |
| PCV7-Argentina | Giglio ND, Caneb AD, Micone P, Gentile A | Vaccine 2010; 28: 2302–2310 | Static Cohort model (Markov model [base case: without HP; sensitivity analyses with HP]) | Hypothetical birth cohort of 696,451 individuals in Argentina in 2006; (follow up over life time) | 92% | 2007 US dollar |
| PCV10-Peru | Gomez J.A., Tirado J.C., Navarro Rojas A.A., Castrejon Alba M.M., Topachevskyi O. | BMC Public Health 2013, 13:1025 | Static Cohort model (Markov age-compartmental, deterministic model;[base case: without HP; sensitivity analyses with HP]) | Birth cohort in Peru; (follow up over life time) | 95% for PHiD-CV and PCV13; 83% for PCV7 | 2009 US dollar |
| PCV7-Japan | Hoshi, S. L., Kondo, M., & Okubo, I. | Vaccine 2012; 30: 3320–3328. | Static Cohort model (Markov model; [base case: without HP; sensitivity analysis: with HP]) | Birth cohort in Japan; (time frame 5 years) | 80% | 2011 Japanese yen |
| PCV7-Netherlands | Hubben G.A.A., Bos J.M., Glynn D.M., van der Ende A., van Alphen L., Postma M.J. | Vaccine. 2007; 25: 3669-78 | Cohort model (Decision tree analytic model - Monte Carlo simulation; *[base case: includes HP*; additional analyses without HP]) | Birth cohort of 200,000 infants in Netherlands in 2001 | 52%-67% | 2004 euro |
| PCV13, PCV9/PCV10, PCV7-Gambia | Kim SY, Lee G, Goldie SJ | BMC Infectious Diseases 2010; 10:260 | Static Cohort model (Marko model [without HP] aggregate level static transition model-TreeAge Pro 2008; sensitivity analyses: with HP also reported) | Hypothetical Gambian birth cohort of 60,000 individuals; (time horizon 5 years) | 90% (80%-100%) | 2005 US dollar |
| PCV10-Argentina, Peru, Chile, Colombia, Brazil, Mexico | Martí S.G., Colantonio L., Bardach A., Galante J., Lopez A., Caporale J., Knerer G., Gomez J.A., Augustovski F., Pichon-Riviere A. | Cost Effectiveness and Resource Allocation 2013, 11:21 | Static Cohort model; deterministic decision tree compartmental simulation model; (base case: without HP; sensitivity analyses: with HP) | Population under 10 years in 6 Latin American countries (Argentina, Brazil, Chile, Colombia, Mexico, Peru) | 95% | 2008 US dollar for Brazil, Chile, Colombia, Mexico and 2009 US dollar for Argentina, Peru |
| PCV7-UK | McIntosh EDG, Conway P, Willingham J, Hollingsworth R, Lloyd A | Vaccine 2005; 23 1739–1745 | Cohort model (*base case: with 32% HP*; sensitivity analyses: without HP) | UK birth cohort; (10 year time horizon) | 95% | 2002 Great Britain pound |
| PCV7-England-Wales | Melegaro A, Edmunds W.J. | Vaccine 2004; 22:4203–4214 | Static Cohort model (base case: without HP; sensitivity analyses: with HP) | Two hypothetical birth cohorts one vaccinated and one unvaccinated; (follow up over life time) | Not reported | 2002 Great Britain pound |
| PCV13, PCV10, PCV7-Australia | Newall AT, Creighton P, Philp DJ, Wood JG, MacIntyre CR | Vaccine 2011; 29:8077– 8085 | Static Cohort model (Static-deterministic state transition model;[base case: without HP; sensitivity analysis: with HP]) | Birth cohort of 300,639 individuals in Australia in 2009; (model with 100 years follow up) | 75%, 93%, 95% (1^st^, 2^nd^ and 3d year of life) | 2009 Australian dollar |
| PCV7-USA | Ray GT, Whitney CG, Fireman BH, Ciuryla V, Black SB | Pediatr Infect Dis J 2006; 25: 494–501 | Population Decision analysis model (*base case: with HP for IPD*; sensitivity analysis: without HP) | Entire US population (including new cohorts of infants); (5 year time frame) | 70% | 2004 US dollar |
| PCV13, PCV7-USA | Rubin JL, McGarry LJ, Strutton DR, Klugman KP, Pelton SI,  Gilmore KE, Weinstein MC | Vaccine. 2010;28: 7634-43 | Cohort model; (Decision-analytic Markov state-transition model; [*base case: with HP for children <2 yrs*; sensitivity analyses: without HP and with broader HP]) | US birth cohort; (10 year time horizon) | 90% | 2008 US dollar |
| PCV13, PCV10, PCV7-Singapore | Tyo KR, Rosen MM, Zeng W, Yap M, Pwee KH,  Ang LW, Shepard DS | Vaccine. 2011; 29: 6686-94 | Cohort model; (Markov model; *[Base case: with HP 20% of direct effect;* sensitivity analyses without HP]) | Infant & child cohort of 226,000 individuals in Singapore | 95% (3 doses) | 2010 US dollar |
| PCV13, PCV10-Argentina | Uruena A, Pippo T, Betelu MS, Virgilio F, Giglio N, Gentile A, Jimenez SG, Jáuregui B, Clark AD,Diosque M, Vizzotti C | Vaccine 2011; 29: 4963– 4972 | Static Cohort model; TRIVAC decision-support model from Pan American Health Organizations’ PanVac Initiative ; (base case: without HP; sensitivity analyses with HP) | 20 successive birth cohorts in Argentina; (follow up for 5 years) | 99% (maximum coverage reached) | 2009 US dollar |
| PCV7-Brazil | Vespa G., Constenla DO, Pepe C, Safadi MA, Berezin E, de Moraes JC, Herreiras de Campos CA, Araujo DV, de Andrade AL SS | Rev Panam Salud Publica. 2009; 26: 518–28 | Static Cohort model; Decision analysis model; (base case: without HP; sensitivity analysis: with HP) | Hypothetical birth cohort in Brazil; (follow up 5 years) | 96% | 2006 US dollar |
| PCV7-Norway | Wisløff T, Abrahamsen TG, Riise Bergsaker MA, Løvoll O, Møller P, Pedersen MK, Kristiansen IS | Vaccine 2006; 24:5690–5699 | Static Cohort model; (Decision analytic Markov model; [base case: without HP; sensitivity analyses: with HP]) | Birth cohort in Norway; (follow up over life time) | Not reported | 2004 euro |
| Men B-UK  (MenB at 1,4,6,and 12 mo) | Christensen H., Hickman M., Edmunds W.J., Trotter C.L. | Vaccine 2013; 31: 2638– 2646 | A Static Cohort-Markov model  AND a *Transmission Dynamic model* (Susceptible-Infected-Susceptible model) | Birth cohort in England (2008); (follow up over life time) | 91% (for routine vaccination); variable by age (for 1-17 yrs catch up) | 2008 Great Britain pound |
| Men C-Canada  (MenC mass immunization 6 m-20 yrs during outbreak) | De Wals, P., & Erickson, L. | Vaccine 2002; 20: 2840–2844. | Static Population cohort (details for model not reported; base case: without HP; sensitivity analysis: with HP) | All population in Quebec 6 months-20 yrs (1992-1993) | Not reported | 1993 Canadian dollar |
| Men ACWY, MenC-Netherlands | Hepkema H., Pouwels K.B., van der Ende A., Westra T.A., Postma M.J | PLoS ONE 2013; 8: e65036. | Static Decision tree analytic cohort model (analyses without and with HP for Men AWY were reported) | Birth cohort of 185,000 Dutch infants; (time horizon: 99 yrs) | 96% (at 14 months); 94% at 12 years | 2011 euro |
| MenC-England&  Wales | Trotter, C. L., & Edmunds, W. J. | Med Decis Making2006; 26:38–47. | *Dynamic transmission model*, multiple birth cohorts (base case: with HP; sensitivity analysis: without HP) | 75 birth cohorts; (for each simulation the model run for 100 years) | 89% | 2000 Great Britain pound |
| R- All GAVI, Central/South America, EU, Africa, Eastern Mediternean, SE Asia, West Pacific | Atherly, D. E., Lewis, K. D., Tate, J., Parashar, U. D., & Rheingans, R. D. | Vaccine2012; 30: Suppl 1, A7–14. | Static Cohort model; Decision analytic model (Monte Carlo simulations); (base case: without HP; sensitivity analysis: with HP) | Annual birth cohort; (follow-up 5 years) | 90% (60%-100%) | 2010 US dollar |
| RotaTeq-England & Wales | Atkins, K. E., Shim, E., Carroll, S., Quilici, S., & Galvani, A. P. | Vaccine 2012; 30:6766–6776. | *Dynamic model*  AND Static cohort model | Birth cohort of 708,500 UK children; (time horizon: 50 years) | 95% | Average value of 2010 and 2011 Great Britain pound |
| Rotarix-Turkey | Bakir, M., Standaert, B., Turel, O., Bilge, Z. E., & Postma, M. | Vaccine2013; 31:979–986. | Static Cohort model; deterministic Markov model (advanced model for probabilistic sensitivity analyses, including without and with HP) | Birth cohort in Turkey; (follow up: 5 years) | 95%-100% | US dollar (year not specified) (considered 2012) |
| R-Netherlands | Bruijning-Verhagen P., Mangen M.J.J., Felderhof M., Hartwig N.G., van Houten M., Winkel L., de Waal W. J., Marc JM Bonten M.J.M. | BMC Medicine 2013; 11:112 | Static, age-structured, discrete time-event, stochastic multi-cohort model; (base case: without HP; sensitivity analyses: with HP) | Dutch birth cohort of 180,000 infants; (time horizon: 20 years) | 88% [base case-scenario] (65% worst-case scenario-97% best-case scenario) | 2011 euro |
| Rotarix, RotaTeq-Belgium, England & Wales, Finland, France, Netherlands | Jit, M., Bilcke, J., Mangen, M. J., Salo, H., Melliez, H., Edmunds, W. J., Yazdan, Y., et al. | Vaccine2009; 27: 6121–6128. | *Dynamic Cohort model (age structured) (with HP*)  AND Static model (without HP) | Vaccinated and Unvaccinated cohorts of children over the first 5 years of life in Belgium, England and Wales, Finland, France and Netherlands | Vaccine coverage based on HIB and DTPa vaccine coverage in BE: 98%; EW:95% and based on expert opinion in FI: 97%; NL: 97% and FR 75% | 2006 euro |
| Rotarix, RotaTeq-Netherlands | Mangen, M. J., Van Duynhoven, Y. T., Vennema, H., Van Pelt, W., Havelaar, A. H., & De Melker, H. E. | Vaccine 2010; 28: 2624–2635. | Population Stochastic simulation model (allows for coupling with a *dynamic model)* | Whole Dutch population; (time horizon: 20 years) | 97% | 2006 euro |
| R-Netherlands | Rozenbaum, M. H., Mangen, M. J., Giaquinto, C., Wilschut, J. C., Hak, E., & Postma, M. J. | BMC Public Health2011; 11:462. | Static Cohort model (CoRoVa model) (hypothetical cohort-age structured); (base case: without HP; scenario analyses: with HP for children <5 years) | Hypothetical birth cohort of 180,000 Dutch children; (time horizon: 5 years) | 95% | 2010 euro |
| RotaTeq-Netherlands | Tu, H. A., Rozenbaum, M. H., De Boer, P. T., Noort, A. C., & Postma, M. J. | BMC Infect Dis 2013; 13: 54. | Static Cohort simulation model (CoRoVa model; update of Rozenbaum 2011 model); (base case: without HP; scenario analysis: HP for individuals < 5 years and also >5 years) | Hypothetical birth cohort of 180,000 Dutch children | 95% | 2010 euro |
| F-USA (universal flu mass vaccination) | Clements, K. M., Chancellor, J., Nichol, K., DeLong, K., & Thompson, D. | Value Health 2011; 14:800–811 | Static Population-Decision tree model ; (base case: without HP; alternative scenario: with HP) | US population age-stratified (to model age-specific vaccine coverage and vaccine efficacy); (time horizon: individuals lifetime) | Universal mass vaccination high risk/low risk groups: <5 yrs: 62% vs 44%;  5-17 yrs: 47% vs 30%;  18-49 yrs: 45% vs 31%; 50-64 yrs: 62% vs 48%; >65 yrs: 83% vs 71% | 2008 US dollar |
| F-TIV-Australia | Newall A.T., Dehollain J.P., Creighton P., Beutels P.  James G. Wood | PharmacoEconomics 2013;31:693–702 | Static Population model; age-stratified Susceptible Exposed Infectious Recovered (SEIR) model; (base case: without HP; sensitivity analysis: with HP) | Australian population, stratified into 50year age intervals; (time horizon: single year) | Assumptions for vaccine uptakes: 40% and 60% | 2010 Australian dollar |
| F-TIV, LAIV-England & Wales | Pitman, R. J., Nagy, L. D., & Sculpher, M. J. | Vaccine2013; 31:927–942. | *Dynamic transmission model* (age-stratified) | Population in England and Wales; (time horizon: 200 years) | 50% (2-18 years old) | 2008 Great Britain pound |

**Footnotes:** *In all included studies: a) if in the base case scenario, herd immunity was not included, additional subgroup/ sensitivity/scenario/sub-model analyses with herd immunity were also reported; b) if in the base case scenario herd immunity was already included [e.g. dynamic models]; additional analyses without herd immunity were also reported.

**Abbreviations:** HP: Herd Protection; HI: Herd Immunity; P= pneumococcal conjugate vaccines, M= mening­ococcal conjugate vaccines, R=rotavirus vaccines, F=influenza vaccines

**Table D**: ICER-outcome Analyses

| **ICER-Differences: Without-With Herd protection** | **Median (IQR, range)** | |
| --- | --- | --- |
| Differences in ICERs (per-QALYs) | $15,619 (IQR: $ 877 to $ 48,376; range: $26,834 to $422,085 | |
| Differences in ICERs (per-LYs) | $54,871 (IQR: $787 to $115,026; range $-12,719 to $246,657) | |
| Differences in ICERs (per-DALYs) | $49 (IQR: $15 to $1,636; range $5 to $13,581) | |
| **More Favorable Results With Herd Protection** | **88/99 (89%) of ICER-outcome analyses**  (in the remaining 11, the results were already cost saving, even without herd protection) | |
| ICERs (per-QALYs) | 47/55 (85%) of ICERs per-QALYs analyses | |
| ICERs (per-LYs) | 24/27 (89%) of ICERs per-LYs analyses | |
| ICERs (per-DALYs) | 17/17 (100%) of ICERs per-DALYs analyses  (In 10/17 analyses the differences were <$100) | |
| **Subgroup Analysis for ICERs that were Not already cost saving without Herd Protection (N=83 ICER-outcome analyses)** |  | |
| **More Favorable Results With Herd Protection** | **83/83(100%) ICER-outcome analyses** | |
| ICERs (per-QALYs) | 43/43 | |
| ICERs (per-LYs) | 23/23 | |
| ICERs (per-DALYs) | 17/23 | |
| **Subgroup Analysis for ICERs that were already cost saving without Herd Protection (N=16 ICER-outcome analyses)** |  | |
| **More Favorable Results With Herd Protection** | **5/16 (31%) ICER-outcome analyses** | |
| ICERs (per-QALYs) | 4 | |
| ICERs (per-LYs) | 1 | |
| ICERs (per-DALYs) | 0 | |
|  |  | |
| **Target-Vaccination strategy vs no Vaccine (N=79 ICER-outcome-analyses)** |  | |
| **Above the Cost-Effectiveness Threshold**  ($50,000 threshold for more developed countries, and X3GDP/capita WHO-cost-effectiveness threshold for less developed countries)  **Without Herd Protection**  AND had compared a target-vaccination strategy vs no vaccine (N=38) | **38/79 (48%) ICER-outcome-analyses** | |
| ICERs (per-QALYs) | 19 |  |
| ICERs (per-LYs) | 15 | (14 for More Developed countries and 1 from Less Developed Country [Brazil]) |
| ICERs (per-DALYs) | 4 |  |
| **Crossed Below the Cost-Effectiveness Threshold**  ($50,000 threshold for more developed countries, and X3GDP/capita WHO-cost-effectiveness threshold for less developed countries)  **With Herd Protection**  - for those ICER-Analyses that were Above this Threshold Without Herd Protection AND had compared a target-vaccination strategy vs no vaccine (N=38) | **17/38 (45%) outcome analyses** | |
| ICERs (per-QALYs) | 9 | (9 for More Developed countries) |
| ICERs (per-LYs) | 8 | (8 for More Developed countries) |
| ICERs (per-DALYs) | 0 |  |
| **Subgroup Analyses** | **More-Developed Countries** | **Less-Developed Countries** |
| **Crossed Below the Cost-Effectiveness Threshold**  ($50,000 for more-developed or X3GDP for less-developed countries)  **With Herd Protection**  - for those ICER-Analyses that were Above this Threshold Without Herd Protection  AND had compared a target-vaccination strategy vs no vaccine | 17 | 0  (28/29 ICER-analyses in Less Developed countries were already below the Cost-Effectiveness threshold (X3 GDP/capita) for Less Developed Countries-  even Without Herd Protection) |
|  | **Industry involvement** | **No Industry involvement** |
| **Crossed Below the Cost-Effectiveness Threshold**  ($ 50,000 for more-developed or X3GDP for less-developed countries)  **With Herd Protection**  - for those ICER-Analyses that were Above this Threshold Without Herd Protection  AND had compared a target-vaccination strategy vs no vaccine (N=38) | 8/16 (50%) | 9/22 (41%) (p=0.58) |
|  | **Healthcare Perspective** | **Societal perspective** |
| **Crossed Below the Cost-Effectiveness Threshold**  ($ 50,000 for more-developed or X3GDP for less-developed countries)  **With Herd Protection**  - for those ICER-Analyses that were Above this Threshold Without Herd Protection AND had compared a target-vaccination strategy vs no vaccine (N=38) | 10/22 (45%) | 7/16 (44%) (p=0.92) |
|  | **Static model** | **Dynamic transmission model** |
| **Crossed Below the Cost-Effectiveness Threshold**  ($ 50,000 for more-developed or X3GDP for less-developed countries)  **With Herd Protection**  - for those ICER-Analyses that were Above this Threshold Without Herd Protection AND had compared a target-vaccination strategy vs no vaccine (N=38) | 13/24 (54%) | 4/14 (29%) (p=0.13) |
| **Authors Conclusions** |  |  |
| Authors recommended at least one target-vaccine | 24/35 (69%) of the studies |  |
| The target-vaccination strategy could have been cost-Effective under certain assumptions (including herd protection) | 6/35 (17%) of the studies |  |
|  | **Industry funded** | **Non-Industry** |
| The target-vaccine was clearly recommended | 20/24 (83%) of industry funded studies | 6/11 (55%) of studies (p=0.07) |

**Table E:** Table of ICERs (per-QALYs; per-LYs and per-DALYs) Without and With Herd Protection (HP) and Respective Differences with values shown before and after inflation to 2016 US dollars (Corresponding to Table 2).

| Vaccine-Country | Author | Comparator | **Difference**  **ICERs per-**  **QALYs (without vs with HP)** | ICERs per-QALYs Difference (Inflated to USD 2016) | ICERs per-QALYs  Without HP | ICERs per-QALYs  Without HP (Inflated to USD 2016) | ICERs per-  QALYs  With HP | ICERs per-  QALYs  With HP  (Inflated to USD 2016) | **Difference ICERs per-LYs (without vs with HP)** | DIfference ICERs per-LYs  Inflated to USD 2016 | ICERs per-LYs Without HP | ICERs per-LYs Without HP (Inflated to USD 2016) | ICERs per-LYs With HP | ICERs per-LYs With HP (Inflated to USD 2016) | **Difference ICERs per-DALYs**  **(without vs with HP)** | Difference ICERs per-DALY  (without vs with HP) (Inflated to USD 2016) | ICERs per-DALY Without HI | ICERs per-DALY Without HP  (Inflated to USD 2016) | ICERs per  DALY With HI | ICERs per  DALY With HP (Inflated to USD 2016) |
| --- | --- | --- | --- | --- | --- | --- | --- | --- | --- | --- | --- | --- | --- | --- | --- | --- | --- | --- | --- | --- |
| PCV7-Sweden | Bergman A et al. | No vaccine | 31,303 | 36,817 | 38,563 | 45,356 | 7,260 | 8,539 | 56,289 | 66,204 | 64,525 | 75,891 | 8,236 | 9,687 |  |  |  |  |  |  |
| PCV7,PCV13-Switzerland | Blank PR et al. | PCV7 (2+1) | 27,217 | 28,108 | 21,607 | 22,314 | -5,610 | -5,794 |  |  |  |  |  |  |  |  |  |  |  |  |
| PCV10, -Canada | Chuck AW et al. | PCV13 | 14,183 | 15,620 | -18,433 | -20,300 | -32,616 | -35,920 |  |  |  |  |  |  |  |  |  |  |  |  |
| PCV10-Canada | Chuck AW et al. | PCV7 (3+1) | -2,725† | -3,001 | -32,848 | -36,175 | -30,123 | -33,174 |  |  |  |  |  |  |  |  |  |  |  |  |
| PCV13-Spain | Díez-Domingo J et al. | No vaccine | 36,151 | 39,955 | 39,906 | 44,105 | 3,755 | 4,150 |  |  |  |  |  |  |  |  |  |  |  |  |
| PCV13-Canada | Earnshaw SR et al. | PCV 10 (2+1) | -11,453^†^ | - 12,454 | -21,749 | -23,650 | -10,296 | -11,196 | -11,688^†^ | -12,709 | -20,642 | -22,456 | -8,954 | -9,736 |  |  |  |  |  |  |
| PCV7-Argentina | Giglio ND et al. | No vaccine |  |  |  |  |  |  | 2,778 | 3,177 | 5,599 | 6,403 | 2,821 | 3,226 |  |  |  |  |  |  |
| PCV10-Peru | Gomez JA et al. | No  vaccine | 961 | 1,062 | 4,500 | 4,974 | 3,539 | 3,911 |  |  |  |  |  |  |  |  |  |  |  |  |
| PCV7-Japan | Hoshi SL et al. | No vaccine | 50,614 | 53,353 | 93,756 | 98,829 | 43,142 | 45,477 | 122,762 | 129,405 | 227,632 | 239,950 | 104,870 | 110,545 |  |  |  |  |  |  |
| PCV7 co-vaccinate-Japan | Hoshi SL et al. | No vaccine | 50,614 | 53,353 | 93,756 | 98,829 | 43,142 | 45,477 | 122,762 | 129,405 | 227,632 | 239,950 | 104,870 | 110,545 |  |  |  |  |  |  |
| PCV7-Netherlands | Hubben GAA et al. | No vaccine | 35,570 | 44,648 | 52,982 | 66,504 | 17,412 | 21,856 | 53,603 | 67,284 | 73,005 | 91,637 | 19,402 | 24,354 |  |  |  |  |  |  |
| PCV7-Gambia | Kim SY et al. | No vaccine |  |  |  |  |  |  |  |  |  |  |  |  | 40 | 49 | 670 | 813 | 630 | 765 |
| PCV9/10-Gambia | Kim SY et al. | No vaccine |  |  |  |  |  |  |  |  |  |  |  |  | 60 | 73 | 490 | 595 | 430 | 522 |
| PCV13-Gambia | Kim SY et al. | No vaccine |  |  |  |  |  |  |  |  |  |  |  |  | 40 | 49 | 410 | 498 | 370 | 449 |
| PCV10-Argentina | Martí SG et al. | No vaccine | 53 | 59 | 3,348 | 3,700 | 3,295 | 3,642 | 698 | 771 | 14,137 | 15,625 | 13,439 | 14,853 |  |  |  |  |  |  |
| PCV10-Brazil | Martí SG et al. | No vaccine | 796 | 877 | 7,089 | 7,807 | 6,293 | 6,930 | 966 | 1,064 | 8,058 | 8,874 | 7,092 | 7,810 |  |  |  |  |  |  |
| PCV10-Chile | Martí SG et al. | No vaccine | 5 | 6 | -230 | -253 | -235 | -259 | -288^†^ | -317 | -3,799 | -4,184 | -3,511 | -3,867 |  |  |  |  |  |  |
| PCV10-Colombia | Martí SG et al. | No vaccine | 135 | 149 | 4,021 | 4,428 | 3,886 | 4,280 | 408 | 449 | 8,336 | 9,180 | 7,928 | 8,731 |  |  |  |  |  |  |
| PCV10-Mexico | Martí SG et al. | No vaccine | 450 | 496 | 4,594 | 5,059 | 4,144 | 4,564 | 715 | 787 | 5,774 | 6,359 | 5,059 | 5,571 |  |  |  |  |  |  |
| PCV10-Peru | Martí SG et al. | No vaccine | 64 | 71 | 2,975 | 3,288 | 2,911 | 3,217 | 215 | 238 | 6,401 | 7,075 | 6,186 | 6,837 |  |  |  |  |  |  |
| PCV7-UK | McIntosh EDG et al. | No vaccine |  |  |  |  |  |  | 44,076 | 58,093 | 50,628 | 66,728 | 6,553 | 8,637 |  |  |  |  |  |  |
| PCV7-UK | Melegaro A et al. | No vaccine | 82,557 | 108,811 | 90,091 | 118,741 | 7,534 | 9,930 | 162,214 | 213,801 | 170,175 | 224,293 | 7,961 | 10,493 |  |  |  |  |  |  |
| PCV10 (3+1)-Australia | Newall AT et al. | PCV 7 (3+0) | 3,208 | 3,546 | 19,130 | 21,143 | 15,922 | 17,597 |  |  |  |  |  |  |  |  |  |  |  |  |
| PCV13 (3+0)-Australia | Newall AT et al. | No Vaccine | 10,763 | 11,896 | 43,773 | 48,379 | 33,010 | 36,483 |  |  |  |  |  |  |  |  |  |  |  |  |
| PCV10 (3+1)-Australia | Newall AT et al. | No Vaccine | 3,177 | 3,511 | 39,719 | 43,898 | 36,542 | 40,387 |  |  |  |  |  |  |  |  |  |  |  |  |
| PCV13 (3+0)-Australia | Newall AT et al. | PCV 7 (3+0) | 15,667 | 17,316 | 26,660 | 29,465 | 10,993 | 12,150 |  |  |  |  |  |  |  |  |  |  |  |  |
| PCV13 (3+0)-Australia | Newall AT et al. | No Vaccine | 15,046 | 16,629 | 67,299 | 74,380 | 52,253 | 57,751 |  |  |  |  |  |  |  |  |  |  |  |  |
| PCV7-USA | Ray GT et al. | No vaccine |  |  |  |  |  |  | 104,500 | 131,170 | 112,000 | 140,585 | 7,500 | 9,414 |  |  |  |  |  |  |
| PCV13-USA | Rubin JL et al. | PCV 7 (4 doses) | 11,752 | 12,942 | -6,901 | -7600 | -18,653 | -20,542 | -2,722^†^ | -2,998 | -19,519 | -21,496 | -16,797 | -18,499 |  |  |  |  |  |  |
| PCV7 (3 doses)-Singapore | Tyo KR et al. | No vaccine | 188,696 | 205,185 | 231,971 | 252,242 | 43,275 | 47,057 |  |  |  |  |  |  |  |  |  |  |  |  |
| PHid-10 (3 doses)-Singapore | Tyo KR et al. | No vaccine | 195,769 | 212,876 | 240,869 | 261,918 | 45,100 | 49,041 |  |  |  |  |  |  |  |  |  |  |  |  |
| PCV13 (3 doses)-Singapore | Tyo KR et al. | No vaccine | 166,891 | 181,475 | 204,535 | 222,408 | 37,644 | 40,934 |  |  |  |  |  |  |  |  |  |  |  |  |
| PCV-10-Argentina | Uruena A et al. | No vaccine |  |  |  |  |  |  |  |  |  |  |  |  | 1,480 | 1,636 | 8,973 | 9,917 | 7,493 | 8,281 |
| PCV-13-Argentina | Uruena A et al. | No vaccine |  |  |  |  |  |  |  |  |  |  |  |  | 1,296 | 1,432 | 10,948 | 12,100 | 9,652 | 10,668 |
| PCV7-Brazil | Vespa G et al. | No vaccine |  |  |  |  |  |  | 46,653 | 54,871 | 69,632 | 81,897 | 22,979 | 27,028 | 1,363 | 1,603 | 2,034 | 2,392 | 671 | 789 |
| PCV7 (4 doses)-Norway | Wisløff T et al. | No vaccine | 54,723 | 68,690 | 174,118 | 218,556 | 119,395 | 149,867 | 196,505 | 246,657 | 386,791 | 485,508 | 190,286 | 238,851 |  |  |  |  |  |  |
| PCV7 (3 doses)-Norway | Wisløff T et al. | No vaccine | 32,336 | 40,589 | 103,227 | 129,573 | 70,891 | 88,984 | 116,908 | 146,745 | 228,841 | 287,246 | 111,933 | 140,501 |  |  |  |  |  |  |
| MenB (3+1)-UK | Christensen H et al. | No vaccine | 123,920 | 136,472 | 302,010 | 332,601 | 178,090 | 196,129 |  |  |  |  |  |  |  |  |  |  |  |  |
| MenB (3+1)-UK | Christensen H et al. | No vaccine | 134,124 | 147,709 | 304,422 | 335,257 | 170,298 | 187,547 |  |  |  |  |  |  |  |  |  |  |  |  |
| MenB (4+1, catch up at 1-4 yrs)-UK | Christensen H et al. | No vaccine | 261,383 | 287,858 | 442,441 | 487,256 | 181,058 | 199,397 |  |  |  |  |  |  |  |  |  |  |  |  |
| MenB (4+1, catch up at 1-17yr)-UK | Christensen H et al. | No vaccine | 383,264 | 422,085 | 537,979 | 592,471 | 154,715 | 170,386 |  |  |  |  |  |  |  |  |  |  |  |  |
| Meningococcal polysaccharide vaccines (ACYW135 or AC)-Canada | De Wals P et al. | no vaccine | 29,481 | 48,376 | 67,495 | 110,753 | 38,014 | 62,377 | 36,463 | 59,832 | 81,459 | 133,667 | 44,996 | 73,834 |  |  |  |  |  |  |
| MenACWY-Netherlands | Hepkema H et al. | MCC (@ 14m) | 247,750 | 261,156 | 512,446 | 540,176 | 264,696 | 279,019 |  |  |  |  |  |  |  |  |  |  |  |  |
| MenACWY-Netherlands | Hepkema H et al. | MenACWY(@14m) | 340,888 | 359,334 | 712,931 | 751,509 | 372,043 | 392,175 |  |  |  |  |  |  |  |  |  |  |  |  |
| MCC-UK | Trotter CL et al. | No vaccine |  |  |  |  |  |  | 20,184 | 27,792 | 25,203 | 34,703 | 5,019 | 6,911 |  |  |  |  |  |  |
| MCC-UK | Trotter CL et al. | No vaccine |  |  |  |  |  |  | 54,489 | 75,029 | 67,704 | 93,225 | 13,215 | 18,196 |  |  |  |  |  |  |
| MCC-UK | Trotter CL et al. | No vaccine |  |  |  |  |  |  | 54,782 | 75,432 | 68,362 | 94,131 | 13,580 | 18,699 |  |  |  |  |  |  |
| MCC-UK | Trotter CL et al. | No vaccine |  |  |  |  |  |  | 17,142 | 23,604 | 23,847 | 32,836 | 6,705 | 9,232 |  |  |  |  |  |  |
| MCC-UK | Trotter CL et al. | No vaccine |  |  |  |  |  |  | 32,319 | 44,502 | 48,827 | 67,232 | 16,508 | 22,731 |  |  |  |  |  |  |
| MCC-UK | Trotter CL et al. | No vaccine |  |  |  |  |  |  | 83,537 | 115,026 | 141,420 | 194,728 | 57,883 | 79,702 |  |  |  |  |  |  |
| Rotavirus vaccine-All GAVI | Atherly DE et al. | No vaccine |  |  |  |  |  |  |  |  |  |  |  |  | 9 | 10 | 42 | 46 | 33 | 36 |
| Rotavirus vaccine-SEAR | Atherly DE et al. | No vaccine |  |  |  |  |  |  |  |  |  |  |  |  | 14 | 15 | 60 | 65 | 46 | 50 |
| Rotavirus vaccine-EUR | Atherly DE et al. | No vaccine |  |  |  |  |  |  |  |  |  |  |  |  | 38 | 41 | 116 | 126 | 78 | 85 |
| Rotavirus vaccine-WPR | Atherly DE et al. | No vaccine |  |  |  |  |  |  |  |  |  |  |  |  | 41 | 45 | 231 | 251 | 190 | 207 |
| Rotavirus vaccine-AMR | Atherly DE et al. | No vaccine |  |  |  |  |  |  |  |  |  |  |  |  | 13 | 14 | 63 | 69 | 50 | 54 |
| Rotavirus vaccine-AFR | Atherly DE et al. | No vaccine |  |  |  |  |  |  |  |  |  |  |  |  | 8 | 9 | 38 | 41 | 30 | 33 |
| Rotavirus vaccine-EMR | Atherly DE et al. | No vaccine |  |  |  |  |  |  |  |  |  |  |  |  | 5 | 5 | 30 | 33 | 25 | 27 |
| Rotavirus vaccine-UK | Atkins KE et al. | No vaccine | 11,963 | 12,809 | 86,087 | 92,178 | 74,124 | 79,368 |  |  |  |  |  |  |  |  |  |  |  |  |
| Rotavirus vaccine-UK | Atkins KE et al. | No vaccine | 11,964 | 12,810 | 54,704 | 58,574 | 42,740 | 45,764 |  |  |  |  |  |  |  |  |  |  |  |  |
| Rotarix-Turkey | Bakir M et al. | No vaccine | -25^†^ | -27 | -12,192 | -12,592 | -12,167 | -12,565 |  |  |  |  |  |  |  |  |  |  |  |  |
| Rotavirus vaccine-Netherlands | Bruijning-Verhagen | No vaccine | 17,686 | 18,643 | 83,835 | 88,372 | 66,149 | 69,728 |  |  |  |  |  |  |  |  |  |  |  |  |
| Rotarix-France | Jit M. et al. | RotaTeq | -14,521^†^ | -17,079 | -35,262 | -41,473 | -20,741 | -24,394 |  |  |  |  |  |  |  |  |  |  |  |  |
| Rotarix-Finland | Jit M. et al. | RotaTeq | -10,371^†^ | -12,198 | -24,889 | -29,273 | -14,518 | -17,075 |  |  |  |  |  |  |  |  |  |  |  |  |
| Rotarix-Netherlands | Jit M. et al. | RotaTeq | -14,520^†^ | - 17,078 | -22,816 | - 26,835 | -8,296 | - 9,757 |  |  |  |  |  |  |  |  |  |  |  |  |
| Rotarix-UK | Jit M. et al. | RotaTeq | -22,816^†^ | - 26,835 | -68,448 | - 80,505 | -45,632 | -53,670 |  |  |  |  |  |  |  |  |  |  |  |  |
| Rotarix-Belgium | Jit M. et al. | RotaTeq | -10,372^†^ | - 12,199 | -18,667 | -21,955 | -8,295 | - 9,756 |  |  |  |  |  |  |  |  |  |  |  |  |
| RotaTeq-Netherlands (HC) | Mangen MJ et al. | No vaccine |  |  |  |  |  |  |  |  |  |  |  |  | 2,868 | 3,373 | 72,802 | 85,626 | 69,934 | 82,253 |
| Rotarix-Netherlands (S) | Mangen MJ et al. | No vaccine |  |  |  |  |  |  |  |  |  |  |  |  | 11,547 | 13,581 | 61,524 | 72,361 | 49,977 | 58,780 |
| Rotarix-Netherlands (HC) | Mangen MJ et al. | No vaccine |  |  |  |  |  |  |  |  |  |  |  |  | 5,730 | 6,739 | 66,546 | 78,268 | 60,816 | 71,528 |
| RotaTeq-Netherlands (S) | Mangen MJ et al. | No vaccine |  |  |  |  |  |  |  |  |  |  |  |  | 2,918 | 3,432 | 67,802 | 79,745 | 64,884 | 76,313 |
| Rotavirus vaccine-Netherlands | Rozenbaum MH et al. | No vaccine | 24,344 | 26,471 | 62,031 | 67,452 | 37,687 | 40,980 |  |  |  |  |  |  |  |  |  |  |  |  |
| RotaTeq-Netherlands | Tu HA et al. | No vaccine | 16,465 | 17,904 | 20,714 | 22,524 | 4,249 | 4,620 |  |  |  |  |  |  |  |  |  |  |  |  |
| Influenza-USA | Clements KM et al. | targeted agesvaccination | 33,803 | 37,227 | -91,764 | -101,059 | -125,567 | - 138,286 | 29,167 | 32,121 | -72,558 | -79,907 | -101,725 | - 112,029 |  |  |  |  |  |  |
| Influenza (TIV)-Australia (HC) | Newall AT et al. | Current practice | 43,369 | 47,159 | 46,658 | 50,735 | 3,289 | 3,576 |  |  |  |  |  |  |  |  |  |  |  |  |
| Influenza (TIV)-Australia (S) | Newall AT et al. | Current practice | 43,351 | 47,139 | 33,734 | 36,682 | -9,617 | -10,457 |  |  |  |  |  |  |  |  |  |  |  |  |
| Influenza (Current practive+TIV[2-4yrs])-UK | Pitman RJ et al. | No vaccine | 4,310 | 4,747 | 3,536 | 3,894 | -774 | -852 |  |  |  |  |  |  |  |  |  |  |  |  |
| Influenza (Current practive+LAIV[2-4yrs])-UK | Pitman RJ et al. | No vaccine | 3,769 | 4,151 | 2,981 | 3,283 | -788 | -868 |  |  |  |  |  |  |  |  |  |  |  |  |
| Influenza (Current practive+TIV[2-10yrs])-UK | Pitman RJ et al. | No vaccine | 7,282 | 8,020 | 6,675 | 7,351 | -607 | -668 |  |  |  |  |  |  |  |  |  |  |  |  |
| Influenza (Current practive+LAIV[2-10yrs])-UK | Pitman RJ et al. | No vaccine | 6,724 | 7,405 | 6,099 | 6,717 | -625 | -688 |  |  |  |  |  |  |  |  |  |  |  |  |
| Influenza (Current practive+TIV[2-18yrs])-UK | Pitman RJ et al. | No vaccine | 9,091 | 10,012 | 8,593 | 9,463 | -498 | -548 |  |  |  |  |  |  |  |  |  |  |  |  |
| Influenza (Current practive+LAIV[2-18yrs])-UK | Pitman RJ et al. | No vaccine | 8,778 | 9,667 | 8,255 | 9,091 | -523 | -576 |  |  |  |  |  |  |  |  |  |  |  |  |

**Footnotes:** Citations for included studies= refs 30-33,40-70. **Abbreviations:** H: healthcare perspective; HP: herd protection; ICERs per-QALYs: number of ICERs per-QALYs analyses per study; ICERs per-LYs: number of ICERs per-LYs analyses per study; ICERs per-DALYs: number of ICERs per-DALYs analyses per study; LAIV: live attenuated influenza vaccine; MCC: meningococcal C conjugate vaccine; Men B: meningococcal B conjugate vaccine; NR: not reported; PCV: pneumococcal conjugate vaccine; PHid10:10-valent pneumococcal conjugate vaccine, conjugated to *Hemophilus influenzae* protein-D; S: societal perspective; TIV: trivalent inactivated influenza vaccine.

† In all cases where the difference without vs with HP was negative, the experimental strategy was already cost-saving without inclusion of indirect effects and with inclusion of indirect effects it was still cost saving (based on absolute cost), although the ratio ICER/QALY with vs without herd immunity was not incrementally more favorable.

**Table F:** Authors’ conclusions regarding the Target-vaccination strategy

| Vaccine (Country) | Author | Citation | Recommendation status |
| --- | --- | --- | --- |
| PCV7-Sweden | Bergman A et al. | Scand J Infect Dis. (2008) | Recommended. “Thus, the health benefits of a national vaccination programme can be achieved within a ‘moderate’ or ‘low’ cost per QALY gained.” |
| PCV13, PCV7-Switzerland | Blank PR et al. | Vaccine (2012) | Recommended. "The national immunisation programmes with PCV13 can be assumed cost saving when compared with the current vaccine PCV7 in Switzerland." |
| PCV13, PCV10, PCV7-Canada | Chuck A et al. | Vaccine (2010) | Recommended. "Increased serotype coverage of the 13-valent vaccine is expected to have a substantial public health and economic impact on infectious disease, when considering direct and indirect effects.” |
| PCV13-Spain | Díez-Domingo J et al. | Vaccine (2011) | Recommended. "A universal PCV-13 vaccination program in the Community of Valencia would be a cost effective intervention from the payer perspective after preventing for pneumococcal infections and for decreasing its associated mortality and morbidity.” |
| PCV13, PCV10-Canada | Earnshaw SR et al. | BMC Infectious Diseases (2012) | Recommended. “Considering the epidemiology of pneumococcal disease in Canada, PCV13 is shown to be a cost-saving immunization program because it provides substantial public health and economic benefits relative to PCV10.” |
| PCV7-Argentina | Giglio ND et al. | Vaccine (2010) | Recommended. “Our analysis predicted that routine vaccination of healthy infants <2 years could … This strategic could be highly cost-effective in Argentina.” |
| PCV10-Peru | Gomez JA et al. | BMC Public Health 2013, 13:1025 | Recommended. "The results of this modeling study predict that PCVs are likely to be a cost-effective strategy to help relieve the epidemiological and economic burden associated with pediatric pneumococcal and NTHi diseases for Peru." |
| PCV7-Japan | Hoshi SL et al. | Vaccine (2012) | Recommended. "When we adopt 3 times of GDP as a criterion, a routine vaccination programme of PCV-7 offered to the birth cohort in Japan is “cost-effective” from the societal perspective and the budget impact to municipality is under ¥11 million (US$137,500) per year. PCV-7 co-vaccinated with other vaccine list on the routine vaccination schedule will make the vaccination programme more cost-effective than vaccinated-alone." |
| PCV7-Netherlands | Hubben GAA et al. | Vaccine (2007) | Recommended. “Our model projects a base-case incremental cost-effectiveness ratio (iCER) of D 14,000 (95% uncertainty interval (UI): 9,800–20,200) per quality adjusted life year (QALY) or D 15,600 (95% UI: 11,100–23,900) per life year gained (LYG). At a willingness to pay of 20,000 per QALY for the Netherlands [25] the PCV7 vaccination program can be considered cost effective with a high probability.” |
| PCV13, PCV9/PCV10, PCV7-Gambia | Kim SY et al. | BMC Infectious Diseases (2010) | Recommended. “Assuming a cost-effectiveness threshold of three times GDP per capita, all PCVs examined would be cost-effective at the tentative Advance Market Commitment (AMC) price of $3.5 per dose.” |
| PCV10-Argentina, Peru, Chile, Colombia, Brazil, Mexico | Martí GS et al. | Cost Effectiveness and Resource Allocation 2013, 11:21 | Recommended. “The incorporation of the 10-valent pneumococcal conjugate vaccine into routine infant immunization programs in Latin American countries could be a cost-effective strategy to improve infant population health in the region.” |
| PCV7-UK | McIntosh EDG et al. | Vaccine (2005) | Recommended " The 7-valent PCV appears to be highly cost effective." |
| PCV7-England-Wales | Melegaro A et al. | Vaccine (2004) | Not recommended (UK/Wales), unless HI is also considered ("In base-case analysis excluding HI: PCV vaccine is not likely to be justified economically; inclusion of HI even with partial SS is likely to render infants vaccination cost-effective) |
| PCV13, PCV10, PCV7-Australia | Newall AT, et al. | Vaccine (2011) | Unclear (Australia)"PHID-CV and PCV13 have potential for ...cost saving...However, assumptions regarding herd protection...changed the relative cost-effectiveness...” |
| PCV7-USA | Ray GT et al. | Pediatr Infect Dis J (2006) | Recommended "IPD herd effects in the nonvaccinated population substantially reduce the cost, and substantially improve the cost-effectiveness, of PCV. The cost-effectiveness of PCV in actual use has been more favorable than predicted by estimate created before the vaccine was licensed.” |
| PCV13, PCV7-USA | Rubin JL et al. | Vaccine (2010) | Recommended. “The model predicts that PCV13 is more effective and cost saving compared with PCV7, preventing 106,000 invasive pneumococcal disease (IPD) cases and 2.9 million pneumonia cases, and saving $11.6 billion over a 10-year period.” |
| PCV13, PCV10, PCV7-Singapore | Tyo KR et al. | Vaccine (2011) | Recommended. “Given these changed inputs, our current estimates of infant vaccination against pneumococcal disease in Singapore find such programs to be moderately cost-effective compared to WHO  thresholds.” |
| PCV13, PCV10-Argentina | Uruena A et al. | Vaccine (2011) | Recommended "Routine vaccination against S. pneumoniae in Argentina would be cost-effective with either PCV-10 or PCV-13.” |
| PCV7-Brazil | Vespa et al. | Rev Panam Salud Publica. (2009) | Recommended "At the current vaccine price, conjugate vaccination could be a cost-effective  investment compared to other options to control childhood diseases. Further analysis is required  to determine whether vaccination at the current price is affordable to Brazil.” |
| PCV7-Norway | Wisløff T, et al. | Vaccine (2006) | Not Recommended/ n "In Norway, governmental guidelines indicate that only interventions with cost per life year of<54,000 euros; 4-dose vaccination is not CE even if decision makers include both HI and indirect costs in their decision." “…This implies that four dose vaccination is not cost-effective even if decision makers includes both herd immunity and indirect costs in their decisions. If three doses offer the same protection as four doses, however, vaccination would be cost-saving when indirect costs are included, but not with only herd immunity. |
| Men B-UK  (MenB at 1,4,6,and 12 mo) | Christensen H et al. | Vaccine 31 (2013) 2638– 2646 | Recommended. "New ‘MenB’ vaccines could substantially reduce disease in England and be cost-effective if competitively priced, particularly if the vaccines can prevent carriage as well as disease." |
| Men C-Canada  (MenC mass immunization 6 m-20 yrs during outbreak) | De Wals P et al. | Vaccine (2002) | Unclear “These economic indices are less favorable than those for current routine immunization programs in Canada, but within the range of those for other common health interventions.” |
| Men ACWY, MenC-Netherlands | Hepkema H et al. | PLoS ONE 8(5): e65036. | Not-recommended: "Routine vaccination with MenACWY is cost-saving. With the current epidemiology, a booster-dose with MenACWY is not likely cost-effective. When herd immunity is lost, a booster-dose has the potential of being cost-effective. A dynamic model should be developed for more precise estimation of the cost-effectiveness of the prevention of disappearance of herd immunity." |
| MenC-England&  Wales  (MCV-C) | Trotter CL et al. | Med Decis Making (2006) | Recommended. The authors found that including herd immunity improved the average cost-effectiveness ratio in all cases, although the extent depended on the vaccine strategy considered. Models that do not include the indirect effects of vaccination will underestimate the impact of MCC vaccination and may lead to distorted decision making.” |
| R- All GAVI, Central/South America, EU, Africa, Eastern Mediternean, SE Asia, West Pacific | Atherly DE et al. | Vaccine (2012) | Recommended. "Rotavirus vaccination in GAVI-eligible countries is very cost-effective and is projected to substantially reduce childhood mortality in this population." |
| RotaTeq-England & Wales | Atkins KE et al. | Vaccine (2012) | Recommended. “Our results indicate that rotavirus vaccination would be beneficial to public health and could be economically sound.” |
| Rotarix-Turkey | Bakir M et al. | Vaccine (2013) | Recommended. "Both projected that rotavirus vaccination in Turkey would improve health outcomes and may result in savings in direct healthcare costs to offset the cost of vaccination." |
| R-Netherlands | Bruijning-Verhagen P et al. | BMC Medicine 2013, 11:112 | Not Recommended. "Universal RV vaccination is the preferred strategy to decrease the high disease burden among young children caused by RV in European countries and elsewhere, but is probably not cost-effective from the healthcare provider perspective. Targeted RV vaccination of high-risk infants is highly cost-effective and can nearly eliminate RV mortality in developed countries with very limited impact on healthcare budgets. We, therefore, encourage policy makers in countries without RV vaccination programs to prioritize RV vaccination for high-risk infants. |
| Rotarix, RotaTeq-Belgium, England & Wales, Finland, France, Netherlands | Jit M et al. | Vaccine (2009) | Recommended (1/5 analyses) and Unclear (3/5 analyses). “Under base case scenario (…no HI)…is likely to be cost-effective in Finland only; incorporating HI...may be CE also in France, Belgium and Netherland” |
| Rotarix, RotaTeq-Netherlands | Mangen MJ et al. | Vaccine (2010) | Not recommended (NL): “Under the current assumptions, only large indirect effects by herd immunity, and/or a far lower vaccine price than the one assumed in the current study would lead to acceptable CUR estimates” |
| R-Netherlands | Rozenbaum MH et al. | BMC Public Health (2011) | Unclear (NL) "Our economic analysis indicates that inclusion of rotavirus vaccination... might be cost effective depending on the cost of the vaccine and the impact of RGE on child's quality of life.” |
| RotaTeq-Netherlands | Tu HA et al. | BMC Infect Dis (2013) | Recommended. "We concluded that the results on potentially favorable cost-effectiveness in the previous study remained valid, however, the new data suggested that previous results might represent an underestimation of the economic attractive.” |
| F-USA (universal flu mass vaccination) | Clements KM et al. | Value Health (2011) | Recommended. "Universal Mass Vaccination against seasonal influenza is cost saving in the US under reasonable assumptions for coverage, cost, and efficacy." |
| F-TIV-Australia | Newall AT et al. | PharmacoEconomics (2013) 31:693–702 | Unclear. "Universal childhood influenza vaccination is likely to be cost-effective if a substantial herd protection effect can be achieved by the program." |
| F-TIV, LAIV-England & Wales | Pitman RJ et al. | Vaccine (2013) | Recommended. “Paediatric vaccination would appear to be a highly cost-effective intervention that directly protects those targeted for vaccination, with indirect protection extending to both the very young and the elderly.” |

**Footnote:** Citations for included studies = Reference 35-69 in main manuscript

**Text A:** PubMed search Strategy (Last search 01/02/2014)

(cost-effective OR cost* [ti] OR cost-effectiveness [ti] OR cost-benefit [ti] OR cost analysis [ti] OR cost-analysis [ti] OR cost utility [ti] OR cost-utility [ti] OR economic evaluation [ti] OR economic analysis [ti] OR economic impact [ti] OR economic [ti]) AND (vaccin* [ti] OR immuniz* [ti] OR immunis* [ti]) AND (meningoc*[ti] OR neisseria [ti] OR pneumococ*[ti] OR rota*[ti] OR influenza [ti]).

**Text B:** WHO-cost effectiveness threshold for Less Developed Countries (as reported in individual studies)

(For less-developed countries with no active comparator (no vaccine), the WHO-cost-effectiveness-threshold of ICER=X3GDP per capita [gross domestic product] was used^36^)^:^

Giglio et al. –Argentina (2007)-: X3GDP per capita threshold= $35,010 US dollars;

Gomez et al.-Peru (2009): X3GDP per capita threshold= $13,068 US dollars;

Kim et al –Gambia (2005): X3GDP per capita threshold= $1,080 US dollars;

Marti et al.

Argentina: X3GDP per capita threshold (2009)= $ 23,990 US dollars;

Brazil: X3GDP per capita threshold (2008)=$ 27,233 UD dollars

Chile: X3GDP per capita threshold (2008)= $34,859 US dollars

Colombia: X3GDP per capita threshold (2008)= $15,954 US dollars

Mexico: X3GDP per capita threshold (2008)= $31,942 US dollars

Peru: X3GDP per capita threshold (2008)= $ 13,662 US dollars

Uruena et al.Argentina (2009): X3GDP per capita threshold= $ 22,098 US dollars

Vespa et al. Brazil (2006): X3GDP per capita threshold=$ 19,232 US dollars;

Atherly et al (GAVI):

All GAVI (2010): X3GDP per capita threshold= $ 8,400 US dollars;

Americas-GAVI (2010): X3GDP per capita threshold=$ 7,800 US dollars;

European-GAVI (2010): X3GDP per capita threshold=$8,400 US dollars;

Africa-GAVI (2010): X3GDP per capita threshold=$ 3,600 US dollars;

East Mediterranean Region-GAVI (2010): X3GDP per capita threshold= $ 3,900 US dollars;

South East Asia-GAVI (2010): X3GDP per capita threshold=$ 5,400 US dollars;

West Pacific Region-GAVI (2010): X3GDP per capita threshold=$ 3,900 US dollars;

Bakir et al.-Turkey (2012): X3GDP per capita threshold= $32,925 US dollars.

**Text C: Prior systematic reviews of CEA studies** for childhood vaccinations

(References at Text E of S1 file)

*Prior Pneumococcal-Conjugate vaccine CEA systematic reviews*

We identified 8 recent systematic reviews of pneumococcal conjugate vaccine CEAs.

Beutels et al (2007)[^6^](#_ENREF_6) identified 15 CEA up to 2006; critically discussed the variation in assumptions across CEA and discussed the impact of HP and/or serotype substitution in 5/15 CEA studies.

Isaacman et al. (2008)[^7^](#_ENREF_7) analyzed 6 pneumococcal-conjugate vaccine CEA published up to 2006 (2 in US, 3 in UK and 1 in Norway) and evaluated the impact of HP. A significant impact of HP was demonstrated.[^7^](#_ENREF_7)

Ray et al (2008)[^8^](#_ENREF_8) analyzed 16 pneumococcal-conjugate vaccine CEA in North America, Australia and Europe and critically reviewed model assumptions. Data with and without HP for ICER per-QALY and ICER per-LY were reported in only 2 studies and 4 studies respectively.[^8^](#_ENREF_8) When indirect effects and QALYs were taken into account, the estimated ICERs were likely to be within the favorable range. [^8^](#_ENREF_8)

Boonacker et al (2011)[^9^](#_ENREF_9) analyzed 21 pneumococcal-conjugate vaccine CEAs up to 2010 against acute otitis media in children and critically reviewed model assumptions and certain ICERs but without focusing on analyses with vs without HP. HP effect was separately discussed in only one study by Ray et al[^10^](#_ENREF_10) included in this systematic review.[^9^](#_ENREF_9)

Farkouh et al (2012)[^11^](#_ENREF_11) in a qualitative systematic review of 16 recent PCV10 and PCV13 CEAs from the past 5 years documented that inclusion of indirect effects was among the three inputs that varied the most across analyses.

Ozawa et al (2012)[^12^](#_ENREF_12) qualitatively reviewed several vaccines in low and middle income countries, including 5 CEA for pneumococcal vaccines. Many CEA in such country-settings used narrow perspectives and did not capture HP benefits and comparative analyses with vs without HP were not reported. [^12^](#_ENREF_12)

Van de Vooren et al (2014)[^13^](#_ENREF_13) analyzed 10 CEA conducted in the European union on the pneumococcal-conjugate vaccines and critically reviewed the model assumptions and types of sensitivity analyses performed across studies and reported the baseline ICERs and conclusions across studies. They concluded that the European CEA studies were mostly based on weak sources of data.[^13^](#_ENREF_13) Only one study based HP assumptions on national data, while 8 had used foreign data or modeling and 1 did not consider HP.[^13^](#_ENREF_13) Conclusions supported the economic utility of pneumococcal vaccines in all studies except for two cases.[^13^](#_ENREF_13) The extensive use of assumptions based on authors’ choices, due to lack of empirical epidemiologic data, led to large within study and between study variability.[^13^](#_ENREF_13)

Wu et al (2015)[^14^](#_ENREF_14) critically reviewed model assumptions, including also HP, and reported the main CEA results without focusing on analyses with vs without HP. Additional systematic reviews were also published for the other studied vaccines.

*Prior Meningococcal -Conjugate vaccine CEA systematic reviews*

We identified 1 systematic review of meningococcal-conjugate vaccine CEAs.

Kauf et al.(2010)[^15^](#_ENREF_15) reviewed 16 CEA for meningococcal conjugate vaccines and identified HP among the problematic areas in the existing literature. Only a minority of economic analyses on meningococcal conjugate vaccines had considered HP and the most efficient method of including HP remains elusive. Comparison across studies was difficult. However, incorporation of indirect vaccine effects was among the factors (along with indirect costs) that substantially improved the vaccine cost-effectiveness.

*Prior Rotavirus CEA systematic reviews*

We identified 4 systematic reviews on rotavirus vaccines CEAs.

Walker et al (2005)[^16^](#_ENREF_16) did a qualitative review of 8 CEA for rotavirus vaccines (7 for the old and 1 for the new), but there were no comparative data with vs without HP. Plosker et al (2011)[^17^](#_ENREF_17) qualitatively described 13 CEA studies and analyzed factors that might have contributed to the large variability in the ICERs values, including model assumptions such as HP; however, the HP impact was qualitatively described and only for a few studies.

Ozawa et al (2012)[^12^](#_ENREF_12) qualitatively reviewed 18 CEA for rotavirus vaccines (among other vaccines) for low and middle income countries, but there were no comparative data with vs without HP.

Karafillakis et al (2015)[^18^](#_ENREF_18) recently published another qualitative systematic review of 24 CEA studies of rotavirus vaccines in Europe up to 2014, nevertheless, comparative data with vs without HP were not reported. They pointed out that future CEA studies will need to demonstrate that indirect vaccine effects in older children are sustained.[^18^](#_ENREF_18)

*Prior Seasonal influenza vaccine CEA systematic reviews*

We identified 5 systematic reviews of CEA for seasonal influenza childhood vaccination. All were qualitative systematic reviews and none reported comparative quantitative data with vs without HP.

An early systematic review by Jordan et al.[^19^](#_ENREF_19) (2006) had identified only 3 CEA, but none of them had included HP.

The qualitative systematic review by Nichol al (2011),[^20^](#_ENREF_20) identified 20 CEA studies on seasonal influenza; however, only a few studies had incorporated indirect vaccine benefits of vaccinating school children and this might have underestimated the benefits of the strategy. The qualitative systematic review by Newall et al (2012)[^21^](#_ENREF_21) also identified 20 economic analyses for influenza (11 of which were conducted in the US); only 9/20 had considered HP, however no comparative data with vs without HP were reported. The authors concluded that many of the factors most influential to cost effectiveness were methodological choices (including for HP) and they pointed out that use of inferences for indirect vaccine effects from observational studies to other settings can be problematic.[^21^](#_ENREF_21) Moreover, while dynamic models may be preferable for estimating indirect effects than static models; their results are highly sensitive to estimated social contacts.[^21^](#_ENREF_21)

Jit et al (2013)[^22^](#_ENREF_22) reviewed 4 systematic reviews on the cost-effectiveness of seasonal childhood influenza vaccination strategies (Newall 2012[^21^](#_ENREF_21); Nichol 2011[^20^](#_ENREF_20); Coleman 2006[^23^](#_ENREF_23) and Savidan 2008[^24^](#_ENREF_24)); however only the systematic reviews by Newall 2012[^21^](#_ENREF_21) and Nichol 2011[^20^](#_ENREF_20) had considered studies with HP, as discussed above. They concluded that among the challenges in estimating the cost-effectiveness of seasonal influenza is indirect herd protection that depended on setting specific variables that are difficult to directly measure.[^22^](#_ENREF_22) They pointed out that there is a need for population-based trial designs, meta-analyses, time series and transmission dynamic models.[^22^](#_ENREF_22)

**Text D: CEA studies reporting additional Indirect Vaccine effect assumptions**

(References at Text E of S1 File)

*Melegaro et al*.[^13^](#_ENREF_47) considered also additional ICER per-QALY and ICER per-LY analyses for net-indirect effects (serotype substitution [SS] +herd protection [HP]) and compared them to analyses with HP only: the respective estimates for PCV7 vs no vaccination in England/Wales with SS+HP were 5- and 6-fold higher than with HP only (ICER per-QALY: 26,683£ vs 5,013£ and ICER per-LY: 30,093£ vs 5,297£) respectively).

*Marti et al^11^* considered also additional analyses for ICER per-QALY and ICER per-DALY for herd protection + indirect effects (serotype substitution) and compared them to analyses with herd protection only for each included country. The inclusion of indirect effects reduced the estimates for all countries. It was unclear if indirect costs were also included in the combined arm -since the inclusion of indirect costs, in a separate analysis, had greater effect on the ICER than the inclusion of HP-. [ICER per-QALY with HP only vs HP + indirect effect: Argentina – $13,439.35 vs. $5,932.31; Brazil $7,092.43 vs. $4535.93; Chile $-3,511.26 vs $-31,980.90; Colombia $7,928.28 vs $4684.11; Mexico $5,059.29 vs. $3,307.09; Peru $6,186.27 vs. $3,132.50]

*Diez-Domingo et al.*[^4^](#_ENREF_29) considered also additional ICER per-QALY analyses for net-indirect effects (SS+HP) and for SS alone and compared them to analyses with HP only for Spain: the respective estimates for PCV13 vs no vaccination in Spain with SS+HP were 2-fold higher than with HP only (5,045€ vs 2,694€).

*Newall et al.*[*^14^*](#_ENREF_45) *and Tyo et al.*[*^17^*](#_ENREF_72) considered also additional ICER per-QALY analyses for SS alone vs HP alone: In Newall et al the respective estimates for PCV7 vs no vaccination in Australia were ~1.5 fold higher than with HP alone (A$92,000 vs A$64,860). In Tyo et al. the ICER per-QALY with SS alone for Singapore were almost 2-fold higher than with HP alone (With SS alone ICER per-QALY for PCV7, PHiD and PCV13 vs no vaccination were $54,868; $57,090 and $48,007; while with HP alone were $21,704; $22,721 and $18,566 respectively).

*Kim et al.*[^10^](#_ENREF_73) considered also additional ICER per-DALY analyses for SS alone and for net-indirect effects (SS+HP) and compared them to analyses with HP alone for Gambia: ICER per-DALY with SS+HP for PCV7, PCV9&10 and PCV13 vs no vaccination were 830$, 550$ and 480$; the respective ICER per-DALY with HP alone were 630$, 430$ and 370$; while the respective ICER per-DALY with SS alone were 3,960$; 1,170$ and 970$.

*Uruena et al*[^17^](#_ENREF_74) considered also additional analyses for ICER per-DALY with serotype substitution alone (SS) for Argentina and the estimates with SS were at least 1.5 time higher than with HP alone.

*Rozenbaum et al*[^25^](#_ENREF_25)^,^[^26^](#_ENREF_26) and *Robberstad et al* ^27^ which were excluded from our analyses reported data only for net-indirect effects (HP+SS) (with vs without), but did not report data separately for HP (with vs without). Specifically, in Rozenbaum et al (Vaccine 2010) for PCV7 vs no vaccination in Netherlands, the ICER per-QALY with vs without net-indirect effects were: were $24,639 vs $106,441 (after transformation of Euros to US$) and the respective ICER per-LY with vs without net-indirect effects were $27,007 vs. $154,146. In Rozenbaum et al (BMJ 2010) for PCV10 (3+1) vs no vaccination in Netherlands, the ICER per-QALY with vs without net-indirect effects were: €52,947 vs €99,151; for PCV13 (3+1) vs no vaccination were €50,042 vs €91,705; for PCV10 (2+1) were €37,891 vs €71,082 and for PCV13 (2+1) were €35,743 vs €66,572. In Robberstad et al for PHiD-10 vs PCV13 in Norway was found to be more cost-effective with or without net-indirect effects; ICER per-LY with vs. without net indirect effects were: 3,135,000 NOK/LYG vs. 8,898,000 NOK/LY respectively.

**Abbreviations:** HP: herd protection; SS: serotype substitution

**Text E: References cited in Text C and Text D of S1 File**

1. Chuck AW, Jacobs P, Tyrrell G, Kellner JD. Pharmacoeconomic evaluation of 10- and 13-valent pneumococcal conjugate vaccines. *Vaccine.* Jul 26 2010;28(33):5485-5490.

2. Earnshaw SR, McDade CL, Zanotti G, Farkouh RA, Strutton D. Cost-effectiveness of 2 + 1 dosing of 13-valent and 10-valent pneumococcal conjugate vaccines in Canada. *BMC infectious diseases.* 2012;12:101.

3. Jit M, Bilcke J, Mangen MJ, et al. The cost-effectiveness of rotavirus vaccination: Comparative analyses for five European countries and transferability in Europe. *Vaccine.* Oct 19 2009;27(44):6121-6128.

4. Bakir M, Standaert B, Turel O, Bilge ZE, Postma M. Estimating and comparing the clinical and economic impact of paediatric rotavirus vaccination in Turkey using a simple versus an advanced model. *Vaccine.* Jan 30 2013;31(6):979-986.

5. Rubin JL, McGarry LJ, Strutton DR, et al. Public health and economic impact of the 13-valent pneumococcal conjugate vaccine (PCV13) in the United States. *Vaccine.* Nov 10 2010;28(48):7634-7643.

6. Beutels P, Thiry N, Van Damme P. Convincing or confusing? Economic evaluations of childhood pneumococcal conjugate vaccination--a review (2002-2006). *Vaccine.* Feb 9 2007;25(8):1355-1367.

7. Isaacman DJ, Strutton DR, Kalpas EA, et al. The impact of indirect (herd) protection on the cost-effectiveness of pneumococcal conjugate vaccine. *Clinical therapeutics.* Feb 2008;30(2):341-357.

8. Ray GT. Pneumococcal conjugate vaccine: review of cost-effectiveness studies in Australia, North America and Europe. *Expert review of pharmacoeconomics & outcomes research.* Aug 2008;8(4):373-393.

9. Boonacker CW, Broos PH, Sanders EA, Schilder AG, Rovers MM. Cost effectiveness of pneumococcal conjugate vaccination against acute otitis media in children: a review. *PharmacoEconomics.* Mar;29(3):199-211.

10. Ray GT, Whitney CG, Fireman BH, Ciuryla V, Black SB. Cost-effectiveness of pneumococcal conjugate vaccine: evidence from the first 5 years of use in the United States incorporating herd effects. *Pediatr Infect Dis J.* Jun 2006;25(6):494-501.

11. Farkouh RA, Klok RM, Postma MJ, Roberts CS, Strutton DR. Cost-effectiveness models of pneumococcal conjugate vaccines: variability and impact of modeling assumptions. *Expert review of vaccines.* Oct 2012;11(10):1235-1247.

12. Ozawa S, Mirelman A, Stack ML, Walker DG, Levine OS. Cost-effectiveness and economic benefits of vaccines in low- and middle-income countries: a systematic review. *Vaccine.* Dec 17 2012;31(1):96-108.

13. van de Vooren K, Duranti S, Curto A, Garattini L. Cost effectiveness of the new pneumococcal vaccines: a systematic review of European studies. *PharmacoEconomics.* Jan 2014;32(1):29-45.

14. Wu DB, Chaiyakunapruk N, Chong HY, Beutels P. Choosing between 7-, 10- and 13-valent pneumococcal conjugate vaccines in childhood: A review of economic evaluations (2006-2014). *Vaccine.* Mar 30 2015;33(14):1633-1658.

15. Kauf TL. Methodological concerns with economic evaluations of meningococcal vaccines. *PharmacoEconomics.*28(6):449-461.

16. Walker D, Rheingans R. Cost-effectiveness of rotavirus vaccines. *Expert review of pharmacoeconomics & outcomes research.* Oct 2005;5(5):593-601.

17. Plosker GL. Rotavirus vaccine RIX4414 (Rotarix): a pharmacoeconomic review of its use in the prevention of rotavirus gastroenteritis in developing countries. *PharmacoEconomics.* Nov 2011;29(11):989-1009.

18. Karafillakis E, Hassounah S, Atchison C. Effectiveness and impact of rotavirus vaccines in Europe, 2006-2014. *Vaccine.* Apr 27 2015;33(18):2097-2107.

19. Jordan R, Connock M, Albon E, et al. Universal vaccination of children against influenza: are there indirect benefits to the community? A systematic review of the evidence. *Vaccine.* Feb 20 2006;24(8):1047-1062.

20. Nichol KL. Cost-effectiveness and socio-economic aspects of childhood influenza vaccination. *Vaccine.* Oct 6 2011;29(43):7554-7558.

21. Newall AT, Jit M, Beutels P. Economic evaluations of childhood influenza vaccination: a critical review. *PharmacoEconomics.* Aug 1 2012;30(8):647-660.

22. Jit M, Newall AT, Beutels P. Key issues for estimating the impact and cost-effectiveness of seasonal influenza vaccination strategies. *Human vaccines & immunotherapeutics.* Apr 2013;9(4):834-840.

23. Coleman MS, Washington ML, Orenstein WA, Gazmararian JA, Prill MM. Interdisciplinary epidemiologic and economic research needed to support a universal childhood influenza vaccination policy. *Epidemiologic reviews.* 2006;28:41-46.

24. Savidan E, Chevat C, Marsh G. Economic evidence of influenza vaccination in children. *Health policy.* May 2008;86(2-3):142-152.

25. Rozenbaum MH, Sanders EA, van Hoek AJ, et al. Cost effectiveness of pneumococcal vaccination among Dutch infants: economic analysis of the seven valent pneumococcal conjugated vaccine and forecast for the 10 valent and 13 valent vaccines. *Bmj.* 2010;340:c2509.

26. Rozenbaum MH, Hoek AJ, Hak E, Postma MJ. Huge impact of assumptions on indirect effects on the cost-effectiveness of routine infant vaccination with 7-valent conjugate vaccine (Prevnar). *Vaccine.* Mar 11 2010;28(12):2367-2369.

27. Robberstad B, Frostad CR, Akselsen PE, Kvaerner KJ, Berstad AK. Economic evaluation of second generation pneumococcal conjugate vaccines in Norway. *Vaccine.* Nov 3 2011;29(47):8564-8574.

**Text F:** Detailed information on reasons for Study-exclusions (expansion of information provided in Figure 1)

**Reasons for Exclusion for 203/469 screened studies (identified in PubMed and Tuft’s CEA Registry)**

**Pneumococcal: n= 65 studies**

Adult vaccinations (n=47), no pertinent ICER data (n=18)

**Meningococcal: n=5 studies**

Adult vaccinations (n=2), no pertinent ICER data (n=3)

**Influenza: n=114 studies**

Adult vaccinations (n=84), no pertinent ICER data (n=30)

**Rotavirus: n=11 studies**

No pertinent ICER data (n=11)

**Combined studies:**

**Pneumococcal & Influenza: n =8**

Adult vaccinations (n=6), no pertinent ICER data (n=2)

**Reasons for Exclusion for 231/266 potentially eligible studies**:

**Pneumococcal: n=74 studies**

Reviews (n=9), no ICER with HP (n=16), no ICER without HP (n=11), no pertinent ICER data (n=37), no ICER with vs. without HP alone (n=1)

**Meningococcal: n=20 studies**

Reviews (n=3), no ICER with HP (n=5), no ICER without HP (n=7), no pertinent ICER data (n=5)

**Rotavirus: n=82 studies**

Reviews (n=3), no ICER with HP (n=56), no ICER without HP (n=1), no pertinent ICER data (n=22)

**Influenza: n=50 studies**

Reviews (n=7), no ICER with HP (n=7), no ICER without HP (n=3), no pertinent ICER data (n=24), adult and children vaccinated (n=7)

**Combined studies:**

Pneumococcal & Meningococcal: n =2 No ICER with HP (n=1), no ICER without HP (n=1)

Pneumococcal & Rotavirus: n = 3 No ICER with/without HP (n=3)
